# Origination of the Split Structure of Spliceosomal Genes from Random Genetic Sequences

**DOI:** 10.1371/journal.pone.0003456

**Published:** 2008-10-20

**Authors:** Rahul Regulapati, Ashwini Bhasi, Chandan Kumar Singh, Periannan Senapathy

**Affiliations:** 1 Department of Biotechnology, Indian Institute of Technology Madras, Chennai, India; 2 Department of Human Genetics, Genome International Corporation, Madison, Wisconsin, United States of America; 3 Department of Bioinformatics, International Center for Advanced Genomics and Proteomics, Nehru Nagar, Chennai, India; NERC Centre for Ecology and Hydrology, United Kingdom

## Abstract

The mechanism by which protein-coding portions of eukaryotic genes came to be separated by long non-coding stretches of DNA, and the purpose for this perplexing arrangement, have remained unresolved fundamental biological problems for three decades. We report here a plausible solution to this problem based on analysis of open reading frame (ORF) length constraints in the genomes of nine diverse species. If primordial nucleic acid sequences were random in sequence, functional proteins that are innately long would not be encoded due to the frequent occurrence of stop codons. The best possible way that a long protein-coding sequence could have been derived was by evolving a split-structure from the random DNA (or RNA) sequence. Results of the systematic analyses of nine complete genome sequences presented here suggests that perhaps the major underlying structural features of split-genes have evolved due to the indigenous occurrence of split protein-coding genes in primordial random nucleotide sequence. The results also suggest that intron-rich genes containing short exons may have been the original form of genes intrinsically occurring in random DNA, and that intron-poor genes containing long exons were perhaps derived from the original intron-rich genes.

## Introduction

Despite intense investigation since the discovery of introns and the split structure of spliceosomal genes nearly 30 years ago [1]–[11], the mechanism of their origin has remained a mystery. The complex spliceosomal machinery is geared towards the linking together of exons and the elimination of introns, which are rarely functional [12], [13]. The rare functional role of introns is also geared mainly towards splicing, during which such functional introns are themselves eliminated [14], [15]. Thus the evolution of introns together with the highly complex spliceosomal machinery to concomitantly eliminate them is perplexing, and the primary molecular mechanisms underlying the origin of introns and the split-gene structure remain unresolved [1], [2], [11].

Evolutionary studies of introns have primarily been based on the Introns-Early (IE) or the Introns-Late (IL) views. The IE view assumes that introns were present in primitive cells, but does not provide a mechanism for their origination [6], [16]–[18]. The IL view assumes that introns were inserted into preexisting contiguously coding genes, without addressing how the contiguous genes first originated [19], [20]. Proponents of both views have focused their work on the phylogenetic distribution of spliceosomal introns, but the results have varied disparately depending upon the particular assumptions and methodologies adopted [1]–[11], [21]–[25]. Moreover, while these studies explore the history of introns across organisms by comparing the ages of different introns, they have not resolved basic questions about their origin. The “exon theory of genes” states that the introns in early genes were sequence spacers that facilitated efficient recombination between short coding regions (exons) that represented protein domains [6], [16], [26], and that this led to the evolution of new genes capable of coding for complex multi-domain proteins. This theory assigns a logical role for introns in abetting evolution subsequent to their origin, but does not offer a mechanism of their origin. In recent years, both IE and IL proponents have suggested that introns present in primitive eukaryotes may have arisen by a process distinct from that underlying the evolution of more recently acquired introns [2], [3], [8], [11], [25]. It is increasingly realized that a fundamental solution capable of explaining the nascent origin of introns in original split-genes may be possible [1], [2], [9].

Here we have applied an innovative bioinformatics approach to investigate the basic mechanism by which the split-structure of spliceosomal genes originated. We present a comprehensive comparative analysis of open reading frame (ORF) length constraints in the genomes of nine species, including a mammal, two invertebrate animals, a plant, a protist, fungi and bacteria. We tested whether the genome sequence and ORF properties of these diverse species corroborate or disprove the random-sequence origin of the split-gene (ROSG) model. The ROSG model posits that the indigenous occurrence of protein-coding genes in primordial random DNA sequences may underlie the origin of the intron and the split structure of original genes [27]. The occurrence of stop codons in random DNA would have been too frequent to allow functional proteins to be encoded. A logical mechanism by which long coding sequences (CDSs) could be achieved would be by the linking together of short coding pieces (exons) occurring within short ORFs and selective ejection of the intervening random sequences (introns). If substantiated, the ROSG model may provide a long sought after mechanism for the origin of introns and the split structure of spliceosomal genes as well as a functional reason for the origin of spliceosomes. Furthermore, the acceptance or rejection of this model has implications on our understanding of the essential relationship between intron-dense and intron-sparse genomes.

### The Model: Possible origin of split-genes from random genetic sequence

The ROSG model proposes that split genes originated from prebiotic primordial genetic sequences [27]. It is based on the assumption that long chemically-synthesized DNA molecules were available in the prebiotic environment and that the genetic code (the codons) was established prebiotically, so that the coding information can be translated into proteins. If codons were assigned prebiotically, then the stop-codons were needed in the origin of protein-coding sequences. It is possible that the general code contained three stop-codons, which then would limit the average ORF length to about 60 bases in random DNA. The origin and evolution of the genetic code may have first involved 12 amino acids representing the original proteins, instead of the 20 found in modern proteins [28]. Our study assumes the genetic code with three stop codons coding for proteins with 20 amino acids, which represents the modern genetic code in majority of current life forms.

The presence of three stop-codons for every 64 codons limits the average ORF length to about 60 bases in random DNA. Then ORFs longer than a few hundred bases would be exceedingly rare. As any coding sequence has to necessarily occur within an ORF, the length of the ORF will restrict its length, and therefore proteins longer than 200–250 amino acids would not be encoded. A mechanism to circumvent this problem and produce long ORFs was necessary in order to create the longer coding-sequences that code for larger proteins integral to living systems. A workable tactic would be to consecutively link the best of the short coding pieces (analogous to modern exons) available at considerable distances in a long random DNA, and remove the intervening nonsense sequences (analogous to introns), and arrive at almost any necessary length of protein-coding sequence.

## Results

### Extremely short ORFs in random DNA due to frequent stop-codons

**Figure 1:**
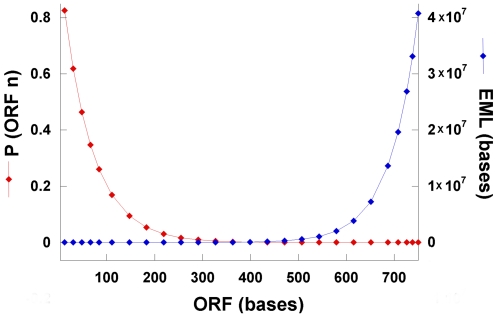
The probability of ORFs of increasing lengths. The probability of ORFs of increasing lengths was calculated based on the formula P (ORF_n_) = (61/64)^n^, where *n* is the ORF length in codons. The Expected Mean Length (EML) for the random DNA for the chance occurrence of ORF*n* is n times the reciprocal of the probability.

While the expected mean length (EML) of the ORFs in a random DNA sequence is 21.3 codons (61 bases), the predicted frequencies of ORFs should decrease exponentially with an increase in length. The probability of the ORFs, and thus the EMLs of random DNA required for the chance occurrence of a given ORF length, are predictable. The probability that a sequence of *n* codons is an ORF is given by P(ORF; *n*) = (3/64)^2^×(61/64)^n^, and for the next occurring ORF in a series of ORFs is given by P(ORF; *n*) = (3/64)×(61/64)^n^. The EML of random DNA required for the chance occurrence of an ORF of *n* codons is *n*/P(ORF; *n*). Figure 1 shows that the probability decreases exponentially and the EMLs increases exponentially as the ORF length increases. This analysis revealed that ORFs longer than 300 bases are extremely improbable in random DNA: for example, 600-base ORFs occur only once in a random DNA length of approximately one million bases, and 750-base ORFs occur only once in 40 million bases.

**Figure 2:**
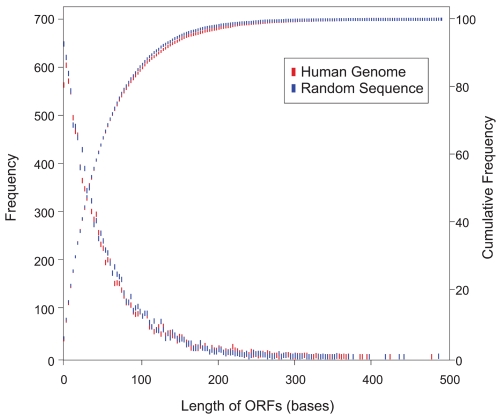
Frequency distributions of ORF lengths in random and eukaryotic DNA. The ORF lengths in all six RFs in a computer generated random DNA sequence (100,000 bases) and in the human genomic sequence [*H. sapiens* chromosome 1, reference: NT_ 004350.18: *base 48,000,000 to base 48,100,000*] were computed and the ORF length frequencies and the cumulative frequencies were plotted.

To test this concept, we plotted the frequency distribution of ORF lengths in a computer generated random DNA sequence of 100,000 bases (Figure 2). The shortest ORF lengths were confirmed to be the most frequent, and the frequency of longer ORFs decreased exponentially, reaching zero at a length of just 300 bases. The majority (∼70%) of the ORFs were shorter than the 60 base EML. The cumulative frequency plot showed that 99% of all ORFs were shorter than 300 bases and 100% of ORFs were shorter than 500 bases. The frequency of ORFs tends to reach zero around a length of 600–750 bases in random DNA billions of bases long, limiting the length of encoded proteins to just ∼250 amino acids. Hence any ORF >750 bases found in an organism's genome can safely be considered as non-random and thus was identified as a non-conforming ORF.

### ORF length constraints in genomes

**Figure 3:**
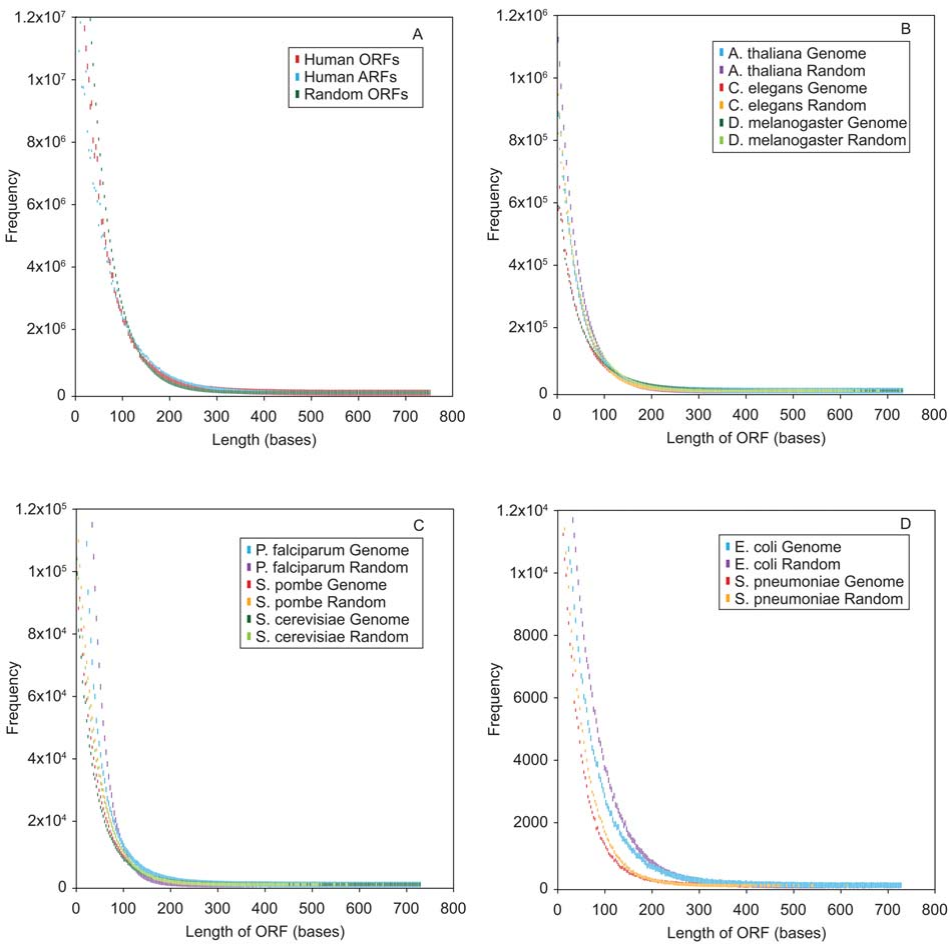
Frequency distributions of ORF lengths in complete eukaryotic and prokaryotic genomes. The ORF lengths in the six RFs of the complete eukaryotic genomes *H. sapiens* (A), *A. thaliana*, *D. melanogaster*, and *C. elegans* (B), *P. falciparum*, *S. pombe* and *S. cerevisia* (C), and the prokaryotic genomes *E. coli* K12 and *S. pneumoniae* R6 (D) were computed and the frequency distributions were plotted. Only frequencies up to 750 bases in length were plotted. The ARF lengths were computed using the amino-acid codons GAC, ACT, CTG (A). The ORF length frequencies were also computed in a computer generated random DNA of length and base composition (See Methods) that matched the respective genomes (B, C, D).

The ORF length frequencies in a stretch of 100,000 bases (Figure 2) from the human genome were strikingly similar to that in random DNA. While ORF frequency distribution from the whole human genome (Figure 3A) was essentially similar to that in random DNA, the vast majority of the ORFs (99.97%) were <750 bases, leaving only a very minute percentage of ORFs (0.027%) that exceeded the 750-base limit (Table 1). Statistical analyses on the data set comprising lengths <750 bases indicated that the human genome sequence had essentially random characteristics (R = 0.9986).

**Table 1:** Frequency of ORFs, Exons, ARFs (between amino-acid codons GTT, ACA, and CTG) & R-ORFs (ORFs computed from a Random Sequence) of increasing lengths in different genomes.

Organism (length of genome in million bases)	Average no. of introns per gene ^1^	Frequency of Exons, ORFs, ARFs & R-ORFs (in *percentages*)					
		< = 750 bases	750–1500 bases	1500–2250 bases	2250–3000 bases	>3000 bases	
*H. sapiens* (3300)	7.4	97.73	1.63	0.38	0.15	0.12	**Exons**
		99.97	0.02	0.002	0.0005	0.0003	**ORFs**
		99.99	0.002	0.0002	3×10^−5^	7×10^−6^	**ARFs**
		99.99	1×10^−5^	0	0	0	**R-ORFs**
*C. elegans* (100)	5	97.75	1.87	0.24	0.06	0.08	**Exons**
		99.95	0.04	0.004	0.002	0.002	**ORFs**
		99.98	0.01	0.002	0.0005	0.0006	**ARFs**
		99.99	7×10^−6^	0	0	0	**R-ORFs**
*A. thaliana* (120)	6.5	94.65	4.12	0.91	0.24	0.07	**Exons**
		99.91	0.07	0.015	0.004	0.002	**ORFs**
		99.99	0.003	0.0001	1×10^−5^	2×10^−5^	**ARFs**
		100	0	0	0	0	**R-ORFs**
*D. melanogaster* (118)	3	87.08	9.58	2.23	0.64	0.47	**Exons**
		99.88	0.09	0.02	0.006	0.007	**ORFs**
		99.99	0.003	0.003	5×10^−5^	0.0001	**ARFs**
		99.99	5×10^−5^	0	0	0	**R-ORFs**
*P. falciparum* (22)	1	69.01	12.32	6.01	3.86	8.8	**Exons**
		99.87	0.06	0.02	0.015	0.04	**ORFs**
		99.91	0.09	0.004	9×10^−5^	0.0007	**ARFs**
		100	0	0	0	0	**R-ORFs**
*S. pombe* (12.5)	1	67.34	17.81	8.42	3.14	3.3	**Exons**
		99.77	0.12	0.06	0.02	0.02	**ORFs**
		99.99	0.006	0.0002	0.0004	8×10^−5^	**ARFs**
		100	0	0	0	0	**R-ORFs**
*S. pneumoniae* (2.2)	0	51.32	37.17	7.88	2.3	1.3	**Exons**
		99.54	0.35	0.08	0.02	0.01	**ORFs**
		99.99	0.01	0	0	0	**ARFs**
		100	0	0	0	0	**R-ORFs**
*E. coli* (5)	0	43.05	43.14	9.58	2.93	1.3	**Exons**
		99.18	0.63	0.13	0.04	0.02	**ORFs**
		99.99	0.003	0	0	0	**ARFs**
		100	0	0	0	0	**R-ORFs**

The lengths between any three of the 61 amino acid codons (amino acid reading-frames, ARFs) in the human genome followed a negative exponential distribution (NED), having far fewer ARFs that were >750 bases in length than the ORFs (Figure 3A and Table 1). This finding was replicated for many three amino acid codon combinations (other than those containing CG dinucleotides, which are deficient in eukaryotic DNA) [27], [29]. Thus the presence of any long ORFs in an otherwise strikingly random human genomic sequence must be the result of some biologically driven process.

We then compared the ORF length distributions in the complete genomes of seven eukaryotic organisms (*Homo sapiens*, *Arabidopsis thaliana*, *Caenorhabditis elegans*, *Drosophila melanogaster*, *Plasmodium falciparum*, *Schizosaccharomyces pombe*, *and Saccharomyces cerevisiae*) and two prokaryotic organisms (*Escherichia coli and Streptococcus pneumoniae*). The genomes were chosen to span a wide-range of intron density: from the highly intron-rich (human), medium intron-rich (Drosophila), intron-poor (Plasmodium and yeast) and intron-less (E. coli) genes. The eukaryotic genomes were also chosen to represent organisms across different phyla: *Homo sapiens* (vertebrata), *Arabidopsis thaliana* (Plantae, Magnoliophyta), *Caenorhabditis elegans* (nematoda), *Drosophila melanogaster* (Arthropoda), *Plasmodium falciparum* (Apicomplexa), *Schizosaccharomyces pombe and Saccharomyces cerevisiae* (Ascomycota).

The frequencies of ORFs <750 bases within all the genomes followed a NED, similar to that in random sequences (Figure 3B, 3C and 3D). Furthermore, >99% of all ORFs in these genomes were <750 bases. The human genome contained the lowest percentage of non-conforming ORFs. The order of the frequencies of ORFs >750 bases among the genomes were as follows: *E. coli* (0.82%)>*S. pneumoniae* (0.46%)>*S. ceresiviae* (0.33%)>*S. pombe* (0.22%)>*P. falciparum* (0.13%)>*D. melanogaster* (0.12%)>*A. thaliana* (0.09%)>*C. elegans*>(0.05%)>*H. sapiens* (0.03%) (Table 1). The ARFs in these genomes also followed a NED similar to that in random DNA. The markedly lesser fraction of ARFs >750 bases relative to ORFs in these genomes indicate that the long non-conforming ORFs in these genomes arose through a selective biological process.

### Non-conforming exons could have originated through intron-loss

Long ORFs must be synthesized from originally random DNA via splicing of nascent exons in order to encode biological proteins. The frequencies of varying lengths of exons and ORFs are shown in Table 1 for eight genomes. The vast majority of exons (97.73%) and ORFs (99.97%) in the human genome did not exceed the length of ORFs in random DNA. Only a small minority (∼2%) of exons were >750 bases, which must be contained within a subset of the 0.027% of non-conforming ORFs. This fraction represents the non-conforming exons that exceed the length predicted for a random sequence.

The frequency of exons <750 bases decreased, while the frequency of ORFs and exons >750 bases increased progressively across widely divergent species in the following order: *H. sapiens*→*C. elegans*→*A. thaliana*→*D. melanogaster*→*P. falciparum*→*S. pombe*→*S. pneumoniae*→*E. coli*. The frequency of non-conforming ORFs in a genome was directly proportional to the frequency of non-conforming exons and increased inversely with the average number of introns per gene. The highly intron-dense human genome had the lowest percentage of non-conforming exons (and ORFs), while the prokaryotes, which contained no introns, had the highest percentage of non-conforming coding sequences analogous to eukaryotic exons (and ORFs).

The fraction of non-conforming exons in the different genomes were proportionate with the fraction of ORFs >750 bases in the corresponding genomes (Table 1). The high congruence of the frequencies of the lengths of the non-conforming exons and the non-conforming ORFs in the different genomes may indicate that the longer ORFs are a direct reflection of increased exon lengths. Because non-conforming ORFs do not occur indigenously in random DNA of any reasonable length, they may be the result of the splicing of two or more exons that existed in ancestral intron-dense genes. Therefore, it is possible that the genomes with the fewest non-conforming exons are closest to the original nascent genomes, and that those with more non-conforming exons may have lost many introns from an ancestral intron-dense genome. Accordingly, the earliest common eukaryotic ancestor may have derived its split genes from random DNA with a full complement of introns, and different organisms might have since lost varying extents of introns. These results are consistent with the observation by Roy & Gilbert [2] that the intron-dense human and *Arabidopsis* genomes resemble the earliest common eukaryotic ancestor, and that introns must have been lost in the relatively intron-sparse genomes. These results perhaps indicate that long exons had evolved by linking together shorter, conforming exons from preexisting genes.

### Striking similarity between split-gene sequences and a random sequence

The ROSG model predicts that the exons must be confined within ORFs, whose lengths must conform to those in random DNA sequences. Thus if split-genes had originated from random DNA, their ORF length distributions should resemble those in random DNA. Therefore, we wanted to compare the ORF distribution of genes from different genomes with those of a random DNA sequence, and analyze whether exons of these genes are confined within the ORFs that conform to a random sequence. For this, we developed a software tool: Exon ORF Plot (EOP, http://66.170.16.154/chandancn/human) (*Username*: *genome*; *password*: *sgopgic*) that plots the occurrence of stop codons and exons of a gene in appropriate reading-frames. Control plots were generated from a random sequence of the same length as each gene, and from an ARF plot of the gene sequence (Figure S1). Examination of human genes revealed that their ORF distribution patterns were remarkably similar to that in random DNA, and that the vast majority of exons (98%) were confined within the short ORFs (sample gene-plots provided in Figure S1), consistent with the ORF and exon length distribution analysis shown in Figure 3. It is possible that the few existing non-conforming ORFs (2%) were perhaps not randomly produced. Instead, they might have been formed by the splicing of ancestral exons. The EOPs demonstrated that 1) the gene ORF plots were highly similar to that of the random DNA, 2) that exons were confined within the short ORFs naturally allowed in random DNA, and 3) exon-splicing specifically created one very long, non-conforming ORF in the spliced gene.

The ORF length distribution in genes from other genomes was also largely consistent with that in random DNA. However, the frequency of non-conforming ORFs that contained long, non-conforming exons were inversely proportionate with the size of the genome (Figure S1). Here too, when exons exceeded 750 bases, the ORFs containing them were clear statistical outliers, suggesting that these were possibly derived from the prior splicing of shorter exons and simultaneous intron loss. In fact, the rare non-random exons were predictably contained within non-conforming ORFs against the background of random ORFs. Importantly, our EOP analyses indicated that the relative incidence of non-conforming exons within very long non-conforming ORFs replicated the order of organisms revealed by the NED analysis above (*S. pombe*>*P. falciparum*>*D. melanogaster*>*A. thaliana*>*C. elegans*>*H. sapiens*).

**Figure 4:**
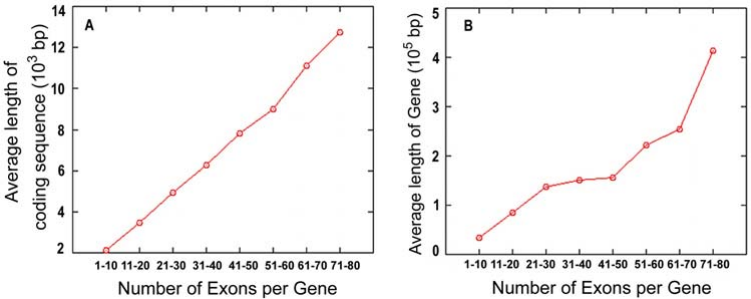
Direct correlation of the number of exons per gene, length of coding sequence, and length of gene. The number of exons, length of coding sequence, and length of gene for each gene in the human genome were tabulated from the EuSplice database [41]. The number of genes with specified number of exons was recorded, and the average lengths of the coding sequence and the gene for this dataset were computed. The average lengths of the coding sequence and the gene were plotted as a function of increasing number of exons per gene up to 80 exons per gene. The figure shows (A) length of coding sequence and (B) length of gene as a function of increasing number of exons per gene.

Does the ROSG model explain extremely long genes with many exons embedded between very long regions of introns? How could they arise from random sequences? Since exons had to be limited in length in random sequence, the longer the coding-sequence of a gene, the more the number of exons it should be split into, and the longer the gene should be. Would this be observed to be true even if the gene was very long? We analyzed this question by tabulating these statistics for all of the genes from the human genome. The results showed that the average number of exons per gene was directly proportional to the average length of the coding sequence per gene, both of which were directly proportional to the average length of the complete split-gene (Figure 4). This was true for genes with small number of exons, and for genes containing large number of exons (e.g., up to 80 exons for which good sample data are available) from the human genome. The average length of exons (approx. 170 bases) and the average length of introns (approx. 4000 bases) were maintained throughout the entire spread.

### The only effect of exon-splicing: A large increase in coding-sequence ORF length

If the ROSG hypothesis is true, then exon splicing in a gene should lead to a significantly long ORF that contains the complete (spliced) protein-coding sequence—a strikingly large length that is well beyond the ORF lengths available within a random genetic sequence. Furthermore, exon splicing should not change the lengths of other ORFs not containing exon sequences or the lengths between any non-stop codons. To test this principle, the exons of a gene were spliced and the stop codons were plotted in the spliced gene sequences (Figure S1). Indeed exon-splicing selectively increased the protein-coding ORF lengths to an extent that does not occur in any length of random DNA (Figure S1). Meanwhile a control study revealed that ARF lengths did not differ before and after splicing (Figure S1). This theme was repeated consistently in nearly all of the human genes, corroborating our conclusion of a specific increase in ORF lengths due to exon splicing (Figure S1).

**Figure 5:**
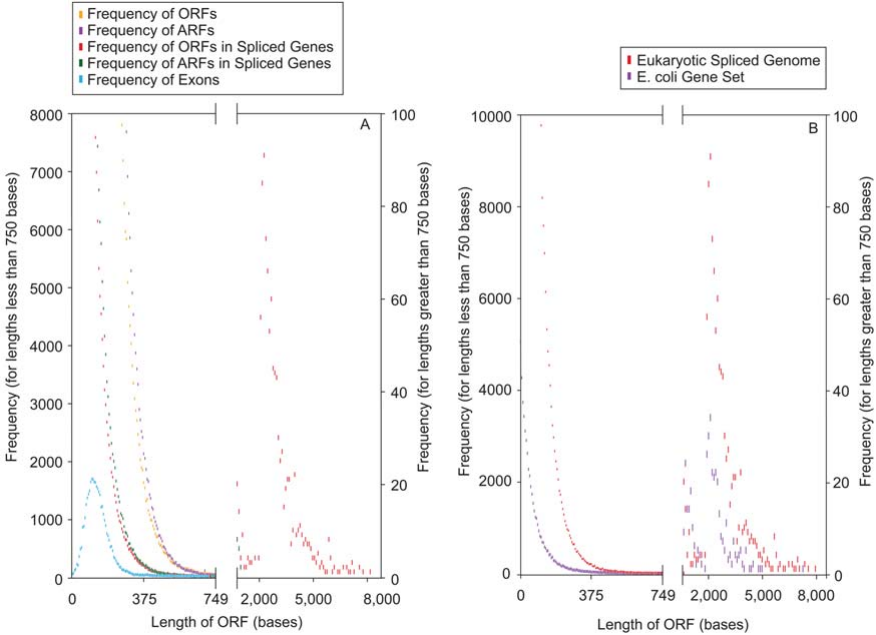
Specific generation of non-conforming ORFs due to exon splicing. (A) Human genes with a complete coding sequence (spliced-exons) >2000 bases and a gene sequence devoid of ORFs >750 bases were selected. The ORF and ARF lengths were computed in all three RFs of each of these genes, and their combined frequencies were plotted. The frequencies of the lengths of exons from this set of genes were also plotted. Next, the exons from each gene were spliced to form its coding sequence and the frequency distribution of ORF and ARF lengths from the spliced sequences were plotted. The X-axis was broken into two parts: from 0–749 bases and from 750–10000 bases. The Y-axis scales corresponding to 0–749 bases are shown on the left, and those corresponding to 750–10000 bases are shown on the right. The frequencies corresponding to 0–749 bases were binned for every 6 consecutive ORF/ARF/exon lengths and the frequencies corresponding to 750–10000 bases were binned every 100 consecutive ORF/ARF lengths. (B) Frequency distribution of ORF lengths in prokaryotic genes. All the genes from the *E. coli* K12 genome, each of whose coding sequence length was at least 2000 bases, were selected. The ORF lengths in all three RFs of each of these genes were computed and their combined frequencies plotted. The ORF length frequency from the spliced sequences of the >2000 base human gene set (Figure 3A) was overlaid for comparison. The methods used for line break, binning and plotting are the same as in Figure 3A.

To study the effects of splicing the exons of genes in a genome-wide experiment, we selected a set of genes from the human genome. To clearly distinguish ORF lengths before and after splicing, we used only genes with a minimum coding sequence length of 2000 bases, and with all the ORFs of each gene being <750 bases in the sense strand. Notably, the vast majority (17,500) of the 27,000 human genes in RefSeq had all of their ORFs <750 bases. Of these, 1,700 had a total coding sequence over 2000 bases. The Negative exponential Distribution (NED) plot of ORFs from this gene set strikingly resembled that from random DNA (Figure 5A). The frequency distribution cure of exon-lengths showed that all of the exons were small enough to be confined to ORFs <750 bases and most (99%) were shorter than 300 bases. The plot from the spliced gene sequences segregated into two distinct sets of ORFs: a curve exhibiting NED approximating random DNA (≤750 bases), and a separate NED curve of non-conforming ORFs ranging from 2000 bases to several thousand bases (Figure 5A). A statistical analysis of the frequencies of ORFs <750 bases showed that this set was highly correlated to that from a random sequence (R = 0.9987). The ARF frequency of the unspliced and spliced genes showed virtually no difference in their overall patterns (Figure 5A), and there was no second ARF NED curve, indicating that the ORF lengths were increased *specifically*. Thus, the only effect of splicing was the generation of a lengthened non-conforming ORF in the spliced sequence relative to the un-spliced sequence in each gene.

### The origin of prokaryotic genes from spliced eukaryotic genes

ORF analysis findings from hundreds of prokaryotic genes were strikingly similar to those of spliced eukaryotic genes (Figure S1). When the ORF length frequency of *E. coli* K12 genes with coding-sequence lengths >2000 bases were plotted (Figure 5B), two distinct clusters of ORF length distribution emerged: the NED pattern with an ORF maximum of ∼750 bases, similar to that in random DNA; and a second cluster of non-random, non-conforming ORFs ranging from 2000 to several thousand bases. This remarkably similar pattern to that of the spliced human genes (Figure 5A) supports the possibility that the long non-conforming ORFs in prokaryotic genes were perhaps derived from the prior splicing of exons from preexisting split genes. Moreover, the ARF pattern from these genes had only one cluster of ARFs (shorter than ∼750 bases), indicating that the longer non-conforming ORFs must have been derived due to a specific biological process during evolution. Thus, while our analysis indicates that the long contiguous protein-coding sequences of extant prokaryotes statistically could *not* have existed in any length of random DNA (see Figure 1), it suggests the possibility that they could be formed only by the linking of originally separate short exons within random DNA. It is possible that the reverse-transcription of the spliced mRNAs of original split-genes could create long contiguously coding genes, which would then precisely exhibit the ORF length characteristics observed in modern prokaryotic genes.

### Genome-wide analysis of non-random ORFs

**Figure 6:**
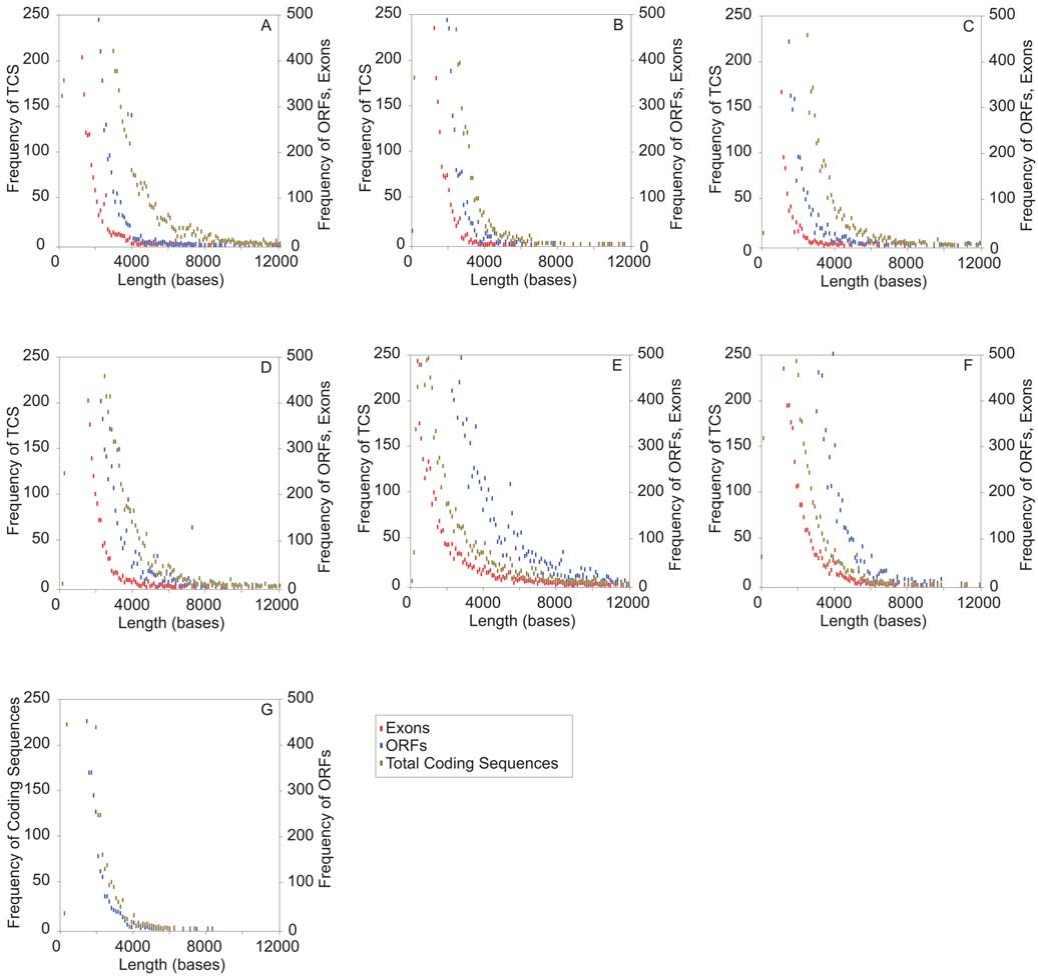
Frequency distributions of the lengths of ORFs, exons, and coding-sequence of genes from different genomes. The frequency distributions of the lengths of exons and the complete coding sequence of genes and the ORFs from the genomes of *H. sapiens* (A), *A. thaliana* (B), *C. elegans* (C), *D. melanogaster* (D), *P. falciparum* (E), and combined fungal genomes of *S. pombe* and *S. cerevisia* (F), and the lengths of the coding sequence of genes and ORFs from the combined prokaryotic genomes of *E. coli* K12 and *S. pneumoniae* R6 (G) were plotted. Frequencies of every 100 consecutive ORF lengths were binned. The frequencies of the TCSs are shown on the left Y-axis and the frequencies of the ORFs and exons are shown on the right Y-axis. In order to magnify the frequencies within the region of non-randomness, the frequencies above certain threshold values (250 and 500 respectively for left and right Y-axes) are not shown, as the frequencies of only ORFs/exons of very short lengths exhibit such large frequencies (Figure 2).

Frequency plots of the lengths of ORFs, exons and the total coding sequence of genes (TCS) in each genome were plotted (Figure 6). Most of the ORFs in the human genome conformed to the random model and were shorter than the respective lengths of the TCS. Note that the exon curves were contained well within the ORF curves. The frequency of non-conforming ORFs and exons increased gradually as one went from the human to the prokaryotes. As expected, the exon curve was almost always contained within the ORF curve. For genomes that clearly conformed to the random model (*Homo*, *Arabidopsis*, *Caenorhabditis*, *Drosophila*) the ORF curve was contained within the TCS curve (Figure 6A, 6B, 6C and 6D), indicating that a high fraction of ORFs were shorter than the TCS. This trend changed at the *Plasmodium*–Fungi “junction” (Figure 6E and 6F), below which the genomes did not conform to the random model, and the ORF curve extended beyond the TCS curve and included ORFs that contained long non-conforming exons; the corresponding exon curves also approached the TCS curves at this junction. Hence this divergence from the random model in protozoan parasite, fungal and bacterial genomes suggested that their long exons were the result of prior splicing of shorter exons from ancestral genes.

### Specific occurrence of stop codons within splice signals

**Figure 7:**
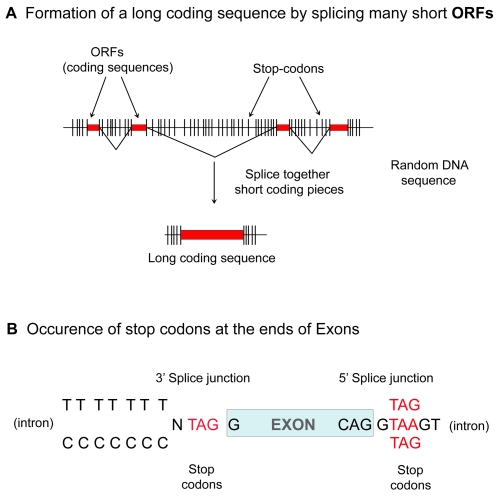
The ROSG model. According to the ROSG model, mRNA splicing evolved to overcome the problem of the frequent occurrence of stop codons in primordial random DNA that severely restricted ORF lengths. (A) Stop codons occurred too frequently to allow functional proteins to be encoded in random DNA. Long contiguous coding sequences were made by the splicing together of short coding-pieces occurring within short ORFs (which became exons) and the elimination of the intervening random sequences (which became introns). (B) Consistent with this model, stop codons are present at exon borders at uniquely high frequencies. The majority of the codons that border the 3′ end of exons are stop codons and all three stop-codons occur at this position (Table S1). Meanwhile one stop-codon (TAG) is predominant at the 5′ end.

According to the ROSG model (Figure 7A), RNA splicing evolved to circumvent the problem of short ORFs caused by frequent stop codons in primordial random DNA. Thus the coding-sequence pieces within the short ORFs became exons, and the intervening sequences with clusters of stop codons became introns. The ROSG model therefore requires that the exons are bordered by stop codons. The EOPs of genes from the seven genomes were examined to verify if this was true (see EOP plots in Figure S1). To further examine this concept, we analyzed the frequencies of the 64 different codons around the splice junctions (at positions −20 to +20 bases with respect to the 5′ and 3′ splice junctions) in all the exons of the human genome. Only the stop codons were found to be present at very high frequencies bordering the exons (Table S1 and Figure 7B). Two key observations were made. Firstly, we found that the majority of the codons (73%) that border the 3′ end of exons (one base after the exons) were stop codons and that all the three stop codons occurred at this position; among the remaining non-stop codons found, the vast majority (∼92%) differed from the three stop codons TAA, TAG, and TGA only by a single base. Secondly, we found that one of the stop codons (TAG) occurred at a high frequency bordering the 5′ end of the exons, and only three other codons (CAG, GAG and AAG) with a single base difference from TAG occurred at this position. The probability for any one of the three stop codons to occur at a position with respect to the splice junction (the +2 position in the donor splice signal) is practically zero, ^(m+n)^C_n_×(3/64)^n^×(61/64)^m^ (where *n* is the number of donor splice signals containing stop codons (200,091) and *m* is the number of donor splice signals with no stop codons (73,336) in the human genome (Text S1). It is even more improbable for stop codons to fortuitously occur on both ends of the exons as observed here. A plausible explanation is that since the exons were selected from ORFs, the stop-codons at exon ends originated from ORF ends within random DNA sequences. Perhaps, these stop codon containing regions later evolved as the splicing signals for supporting the splicing machinery. These observations made from 273,427 exons from the complete human genome are essentially consistent with our previous analysis, which was conducted with a very small number of about 1000 exons [30], [31]. An interesting corollary to this is the fact that splice signal sequences for tRNA and rRNA genes that are not based on protein-coding sequences do not include stop codons [32], [33] – further suggesting that the stop codons at the ends of coding exons may have arisen from the ORF ends in random DNA sequences.

The fact that introns are far longer than exons may be a result of the low probability of the precise combination of splicing signals in random DNA that are required for the elimination of introns. The secondary selection of genes based on the biological usefulness of their encoded proteins could be another reason for this. Our preliminary analysis indicates that, while the split coding sequences for given proteins occur at a far higher probability compared to non-split genes in random DNA sequences, the introns in such genes are far longer than the exons, as typically observed in human genes.

## Discussion

### Random-sequence origin of eukaryotic split coding genes

The initial analysis based on the random-sequence origin of protein-coding genes [27], [30], [31] with limited data provided evidence that the DNA of eukaryotic organisms may be highly random. Given that primordial DNA was long and contained random nucleotide sequence, and the possibility that split coding genes may occur intrinsically within a random genetic sequence, this approach may explain the major characteristics underlying the split-structure of eukaryotic genes. The current study with complete genome sequences strongly corroborates this fundamental theme, and brings out novel insights concerning the randomness of the genome sequences, and the inter-relations between the intron-rich and intron-poor genomes. It shows that the vast majority of genomic sequences (>99%) in all the genomes examined here exhibit random characteristics, and that there exists only a tiny fraction of the ORFs that do not conform to the random model. It is striking that this tiny fraction of ORFs in a genome corresponds and correlates with the fraction of all the long, non-conforming exons in each of the genomes.

Although the current results indicate that the probability of long ORFs occurring in random DNA is small, we found that the smaller genomes of the simpler organisms had a higher frequency of non-conforming ORFs and exons than the larger genomes of the more complex organisms. The study indicates that, because the ORFs and exons are highly restricted in length in a random sequence, a long ORF could only be derived by the splicing together of these short ORFs containing the short exons. If a large genome with intron-rich genes began to lose introns, there would be a drastic reduction in the size of the genome—as approximately 95% of a gene constitutes introns in the large intron-rich genomes such as the human, the content of introns being 20–25 fold higher than that of exons. Simultaneously, such intron loss may also lead to a significant increase in the average length of the exons in the genome. The results here reflect this theme by the reduction of the genome size from the large (human) to the small (Plasmodium) genome, as their average exon length increased, and as the fraction of the non-conforming ORFs increased. Even though the variations of the fraction of non-conforming ORFs among different genomes (human to E. coli) occurred within a tiny range of less than 1%, the increase in the average length of ORFs in the different genomes correlated clearly with the corresponding increase in the average length of exons, which in turn correlated with the reduction in genome size. This result also indicates that a majority of the non-conforming ORFs in each of the genomes was accounted for by the non-conforming exons in the genome.

The present results suggest that the preexistence of long non-random coding sequences in random DNA is exceedingly improbable. Thus, under the ROSG model, prokaryotic genes may not have been possible to occur in a random sequence. The possible conclusion is that introns were present in the most primitive genes, the very first genes were intron-dense, and that the genes of the smaller intron-poor (eukaryotic parasites and yeast) and intron-less (bacteria) genomes were derived from the intron-rich original genes by loss of introns. Our results are supported by the recent findings that introns may have been numerous *before* the divergence of eukaryotes and prokaryotes [2], [3], [5].

The current results suggest that more introns may have been lost over the course of evolution in the genomes which are presently smaller in size, and which have more non-conforming ORFs and exons (Table 1). Interestingly, among the genomes examined here, the human seems to be the closest to random (retaining most of the original introns) and the *E. coli* seems to be the farthest from random. It is remarkable that all the characteristics of random DNA are still essentially present in the split genes of present day intron-dense large genomes such as those in the human. The *Arabidopsis* (120 M bases) and *Caenorhabditis* (100 M bases) genomes are exceptional in that they are relatively small but have high intron densities, owing to their distinctively short introns. It is possible that their introns were originally long, and that they were perhaps shortened during evolution by yet unknown biological processes. This may perhaps mean that the prokaryotic genes may have lost all their introns. It may also mean that the introns present in microbes such as the archeabacteria are the residual remnants of the leftover introns.

The recent discovery of the possible extensive transcription of non-coding RNAs (ncRNAs) from the genome [34] supports the ROSG model. These ncRNAs occur at very high probability in random DNA (data not shown), as most of these RNAs such as the miRNAs and siRNAs are very short. Sequences such as the exon-splice enhancers (that help the removal of introns) within introns are also highly probable in random DNA. Several functional sequences could have fortuitously occurred in these originally non-functional sequences that were useful in the evolution of the genome and the organism. Consistent with the ROSG model, the present study may show that the original reason for the gene to be split was the stop-codon interference problem in a random sequence, and that functional sequences may have fortuitously occurred with high probability within these long non-coding introns (and intergenic sequences) subsequent to their origin in random DNA.

### Consistent multiple evidence for the ROSG model

The current study brings out three major lines of corroborating evidence for the random-sequence origin of protein coding split-genes. First, the very high congruence of the negative exponential distribution of ORF lengths between the intron-rich genomes (e.g., human) and the random DNA sequence (>99.97%, R = 0.9986). Second, the upper limit for the length of exons in intron-rich genomes (that codes for ∼200 amino acid long protein) was found to be the same as the longest coding sequence in random sequence. Third, the distribution of actual exon lengths in intron-rich genomes is congruent with and falls below the distribution of the lengths of random ORFs. The average exon length from the intron-rich genomes is about 170 bases whereas that expected from random ORF lengths is 60 bases. This may indicate that there has been a selection for longer exons within the allowed maximum ORF length of 600 bases for optimizing the frequency of suitable exon lengths. Fourth, the very small fraction of the non-conforming exons (<2%) in intron-rich genomes (e.g., human) are contained within the extremely small fraction of the non-conforming ORFs (0.03%), and a majority of the non-conforming ORFs are filled with non-conforming exons. This phenomenon is observed in every genome examined, even where the fraction of the non-conforming exons increases. Thus the current study may provide mathematical evidence that the long non-conforming ORFs and exons in smaller genomes were derived by splicing short exons from the pre-existing intron-rich genes of larger genomes. The fifth evidence is that the stop-codons that ought to mark out introns do in fact mark them out. The long-observed presence of GURA and YAG at the intron ends has been taken for granted that they ought to be the integral parts of splice junctions, without asking the fundamental question as to why the stop codons that comprise them got to be there in the first place. The current work provides a reasonable mechanism for their origin from the random sequences.

### Origin of the spliceosomal machinery for splicing short ORFs

The ROSG model proposes that protein-coding sequences simply occurred intrinsically in random DNA sequences in a split form that led to the evolution of split-genes for particular proteins. Our other recent research has shown that all of the authentic structures of complex split genes occur intrinsically within random genetic sequences such that an abundant number of split genes were found in a small amount of random DNA (A. Bhasi, B. Balan, B. Kumar and P. Senapathy, Unpublished). Furthermore, numerous split coding genes for complex protein sequences were found to exist intrinsically in random genetic sequences because of the high variability of the amino acid sequences in proteins and the high degeneracy of the codons in coding sequences (P. Senapathy, Unpublished) [36]. If this is the case, a vast number of split genes, each coding for a unique complex protein, could occur indigenously in random genetic sequences. This may enable the trial of different permutations and combinations of genes for various biological structures and functions, and thus pave the way for the evolution of biological systems and possibly complete genomes [36].

Essentially, the ROSG model suggests that split-genes for complex proteins were available in a relatively small amount of random genetic sequences, and these genes could assemble in various combinations to evolve genomes [36]. For example, many complex proteins must have been required for the cellular processes, such as the ribosome or the spliceosome, to have come into existence. These proteins must have been present all at once, or there would have been no meaningful structure of the ribosome or the spliceosome. Recent research shows that almost all of the 110 proteins that constitute the spliceosome must have been present in the eukaryotic ancestor [35]. There has been no organism including the microbes that has any simpler version of the spliceosome. In essence, when the spliceosome came into existence in the first living cell, it must have been as complex as that is found in modern living cells. The ROSG model suggests that this was possible because complete split-genes coding for numerous complex proteins may have simultaneously occurred indigenously in random genetic sequences, from which the correct combination of genes for a particular structure such as the spliceosome was highly probable to be assembled and evolved.

The present findings may suggest a plausible basis for the origin of spliceosomal introns. They indicate that introns may have been the random sequences that needed to be eliminated in order for the coding sequence pieces available within unconnected short ORFs in random DNA to be joined. The spliceosomal machinery may have ultimately evolved to accomplish the ejection of the unneeded introns. The facts that introns rarely exhibit biological function and that the spliceosome functions only to eliminate introns support this interpretation. The results may thus corroborate the ROSG model that the occurrence of the essentially non-coding and useless intron sequences within genes was necessitated due to their origin from random genetic sequences, and that a machinery was required for removing the genetic waste. The results may also suggest that the spliceosomal machinery may have possibly originated within the earliest living cells, which required long functional proteins to be produced from the original genome that contained intron rich split-genes. The recent findings that almost all of the proteins that constitute the modern spliceosome may have been present in the eukaryotic ancestor [35] corroborate the ROSG model's suggestion that the spliceosome necessarily originated within the first eukaryotic cells. The results here may thus explain why introns are essentially non-functional, why they are ubiquitous in eukaryotic genes, why they should have co-originated with the earliest nascent eukaryotic genes, and why they are dispensed off at the expense of the large and complex spliceosomal machinery.

These interesting possibilities are supported by the recent studies of phylogenetic intron distributions, showing that the large intron-dense genomes may have been structurally closer to that of the eukaryotic ancestor [1]–[3]. More recently, several entirely different approaches, based on the comparative genomics of gene content, protein content, protein domain content and the organization of protein domains, have been undertaken for determining the root of the evolutionary tree. These studies have pointed to the intriguing possibility that the genome of the last universal common ancestor (LUCA) of life may have had a complex, eukaryotic-like, gene-rich, sophisticated, and relatively modern organization [37]–[40]. However, these studies have not addressed the fundamental questions: How, when and wherefrom did the genes of the earliest eukaryotic ancestor originate? What was the source and mechanism of the origin of the complex structures of split-genes in the eukaryotic ancestor? The present results based on the ROSG concept may provide credible answers to these questions.

In conclusion, the results show that the spliceosomal genes may have originated indigenously in primordial random genetic sequences, which may explain the origin of both the intervening sequences and the split structure of the original protein-coding genes. This study may provide a reasonable explanation for why the introns are non-coding and non-functional, and why the original genes may have been rich in introns. It also suggests that the large, intron-rich genes containing short exons were perhaps closer to the original genes present in the eukaryotic ancestor, and that the shorter, intron-sparse genes containing relatively longer exons may have been derived by successive loss of introns. This study thus shows the possibility that the genome of the very first living cell may have prebiotically originated from complex split-genes occurring in primordial random genetic sequences—explaining why the LUCA must have been a eukaryote with a complex genome.

## Methods

### Datasets

Reference sequence assemblies of different organisms were downloaded (Jan 2006) from NCBI (ftp://ftp.ncbi.nih.gov/genomes/) in FASTA and GBK formats. All gaps within genome sequences were removed as a preliminary step.

### Generation of ORF and ARF frequencies from the genome and random sequences

Only continuous sequences were extracted from genomes. For each reading frame, the lengths of successive ORFs were computed by counting the number of bases between successive stop codons (TAG, TGA, TAA). This was done in all the 6 frames (3 in each strand). The frequencies of these lengths were plotted as a scatter graph and compared with those from a random sequence. The random sequences were generated by a random base generator that takes into account the predilection given to certain bases in the region under comparison. For example, when a sequence of one million bases from the human genome was compared with that from a randomly generated sequence, the percentage of A, T, G and C within that human sequence was computed, and the random base generator was modified to adjust the weights of these bases while generating them. Corresponding methodologies were used for plotting the frequencies of ARFs (lengths between any three amino-acid codons) from a genome or random sequence. Comparative studies also included random sequences that were generated based on the di-nucleotide composition of a given genome. All plots were drawn with KaleidaGraph 4.02 from Synergy Software. Programs were written in Perl with considerable usage of Bioperl modules.

### Exons and Spliced Genes

Only coding sequences were used for computing the lengths of exons and for simulated splicing. The splicing mechanism was simulated exactly as it happens biologically, by eliminating the intronic regions in between the coding-sequence regions in a particular gene. Once the spliced genes were generated, the pattern of ORFs/ARFs was determined as stated earlier, except that these were scanned only in the ‘sense’ strand. This process was sometimes dynamic (Figure 3A) wherein the genes were spliced only if all ORFs fell below a 750 base limit and the overall length of the spliced gene exceeded a certain limit. These operations were automated to be conducted ‘on the fly’.

## Supporting Information

Text S1 (0.05 MB PDF)

Table S1 (0.06 MB PDF)

Figure S1 (11.77 MB PDF)
