# Supplementary material for: Origination of the Split Structure of Spliceosomal Genes from Random Genetic Sequences
Source: PLoS One. 2008 Oct 20;3(10):e3456. doi: 10.1371/journal.pone.0003456 (PMC2565106; doi:10.1371/journal.pone.0003456)
Supplement: Text S1 — (0.05 MB PDF) [file pone.0003456.s001.pdf]

I. Axis representation and binning of data.

Because the frequencies at small lengths were many times more than the frequencies at longer lengths, the Y axis (corresponding to the frequencies) was sometimes truncated or cut off as mentioned in the figure legends. The computed data were also binned in Fig. 3 and Fig. 5 to eliminate noise within the frequencies without compromising the general trend.

II. Statistical Analysis – Correlation.

Correlational comparisons are reported with the linear correlation coefficient R. The closer this value is to 1.0, the better the two groups being compared are correlated. Correlation coefficient values were calculated using the following formula:

$$\frac{\sum_i (x_i - \bar{x})(y_i - \bar{y})}{\sqrt{\sum_i (x_i - \bar{x})^2}\sqrt{\sum_i (y_i - \bar{y})^2}}$$

III. Binomial probability.

The probability for any of the stop codons to occur at a position with respect to the splice junction (the +2 position in the donor splice signal) was calculated using binomial probability. The general equation for the probability of getting k success out of n trials is

given as ${}^{n}C_{k}\, p^{k}q^{n-k}$, where p is the probability of success and q is the probability of failure ($q = 1 - p$). In an analogous fashion, in a DNA sequence comprising *n*+*m* exons, the probability for any of the stop codons to occur at the *n* donor splice signals and to not occur at the remaining *m* donor splice signals would be ${}^{(m + n)}C_{n} \times (3/64)^{n} \times (61/64)^{m}$.
