# Supplementary material for: Origination of the Split Structure of Spliceosomal Genes from Random Genetic Sequences
Source: PLoS One. 2008 Oct 20;3(10):e3456. doi: 10.1371/journal.pone.0003456 (PMC2565106; doi:10.1371/journal.pone.0003456)
Supplement: Table S1 — (0.06 MB PDF) [file pone.0003456.s002.pdf]

SUPPLEMENTARY TABLE

TABLE S1: High frequencies of stop codons at exon borders in the human genome.

CODON	Acceptor Splice Site (-3 position)		Donor Splice Site (+2 position)	
	Count	Percent	Count	Percent
TGA	13	0.005	76617	28.02
TAA	7	0.003	103427	37.83
TAG	76969	28.15	20047	7.33
CAG	178350	65.23	218	0.08
SBD	16936	6.19	67157	24.56
OTHERS	1147	0.42	5961	2.18
TOTAL	273422	99.99	273424	100

All of the available data for human exons (273,426) were examined.
SBD: codons which differ from stop codons by a single base.
