# Supplementary material for: Origination of the Split Structure of Spliceosomal Genes from Random Genetic Sequences
Source: PLoS One. 2008 Oct 20;3(10):e3456. doi: 10.1371/journal.pone.0003456 (PMC2565106; doi:10.1371/journal.pone.0003456)

SUPPLEMENTARY FIGURE

FIGURE S1: Comparison of the Exon-ORF Plots (EOPs) of representative genes from *H. sapiens*, *C. elegans*, *P. falciparum*, *S. pombe*, and *E. coli* with random DNA ORF plots. The ORF distribution patterns in the three RFs of each gene sequence and its corresponding spliced gene sequence were mapped. The exons and the spliced coding-sequence were overlaid on the appropriate ORF and on the RF in which they occurred. For each gene, the gene details are summarized in a table above the plots. A control reference ORF distribution pattern for a computer-generated random DNA of the same length of each example gene was plotted to enable immediate visual comparison (top plot). The plots shown for each gene are: the ORF plot of the split-gene with overlaid exons (middle left), the spliced gene with overlaid complete coding-sequence (middle right), the ARF plot of the unspliced split-gene (bottom left) and the ARF plot of the spliced-gene sequence (bottom right).

Introduction to the Plots

Representative mammalian (*H. sapiens*) genes

EOPs of 12 representative human genes are displayed on pp. 3-14. We found that human gene sequences were remarkably similar to random DNA, with nearly all (99.97%) of their ORFs (vs. 99.99% of random ORFs) being shorter than 750 bases and 99.13% (vs. 99.83% of random ORFs) being shorter than 300 bases. The ARFs (amino acid reading-frames; the distances between any three non-stop codons) in human genes also exhibited random characteristics. Our analysis revealed that the vast majority of exons in human genes (98%) are shorter than 750 bases, and >92% are shorter than 300 bases. Very rarely did human exons not conform to the random sequence model. Note that the human genes are also highly intron dense, having an average of 8 introns per gene.

Exon ORF Plot for Gene CP

[Genome: Homo sapiens] [Gene Name: ceruloplasmin (ferroxidase)]

Gene ID: **1356** Gene Symbol: CP Gene Length: 48204 Number of Exons: 19

**Exon**	1	2	3	4	5	6	7	8	9	10	11	12	13	14	15	16	17	18	19
**Length**	146	248	213	174	255	172	140	153	212	151	213	208	140	129	107	217	140	163	17

Total Spliced Exon Length: 3198

TAA: | TGA: | TAG: | Exon:

Random Sequence ORF

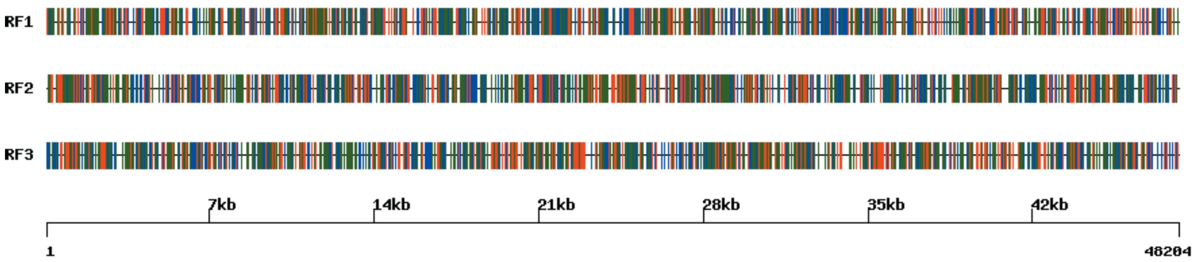

Exon ORF (Before Splicing)

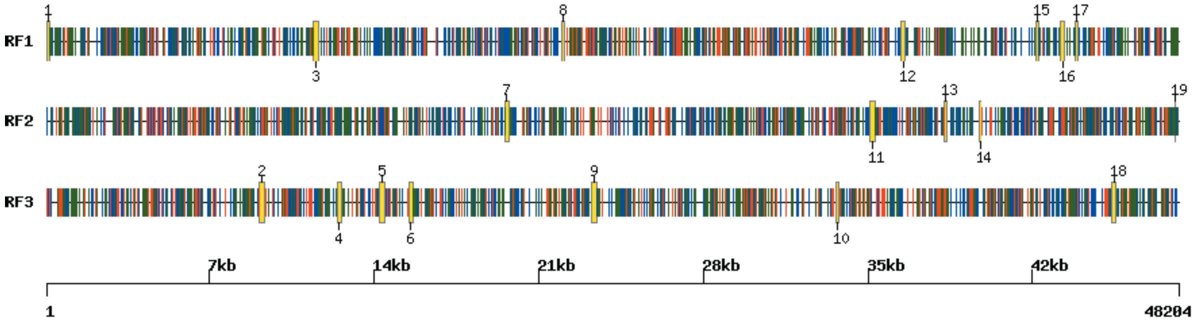

Exon ORF (After Splicing)

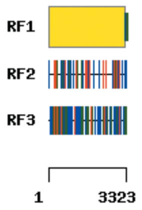

GTT : | ACA : | CTG : |

Exon ARF (Before Splicing)

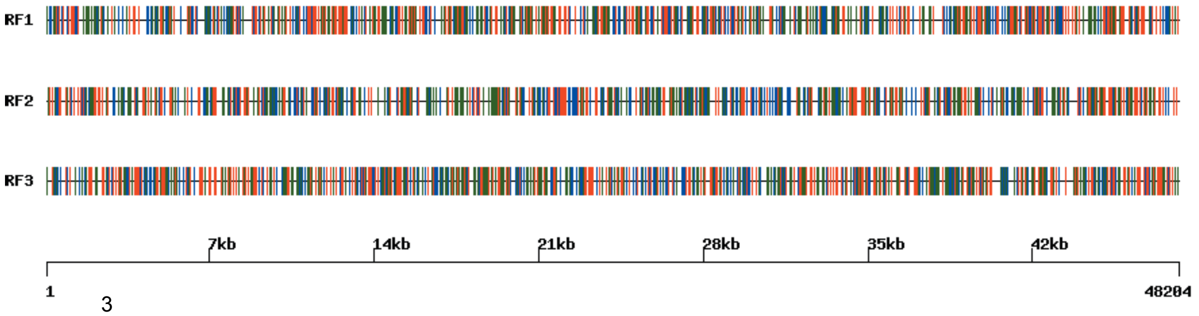

Exon ARF (After Splicing)

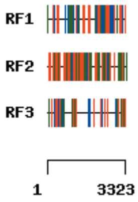

Exon ORF Plot for Gene GALC

[Genome: Homo sapiens] [Gene Name: galactosylceramidase precursor]

Gene ID: **2581** Gene Symbol: GALC Gene Length: 60484 Number of Exons: 17

**Exon**	1	2	3	4	5	6	7	8	9	10	11	12	13	14	15	16	17
**Length**	147	69	64	114	140	39	131	156	125	128	90	87	151	181	164	77	147

Total Spliced Exon Length: 2010

TAA: | TGA: | TAG: | Exon:

Random Sequence ORF

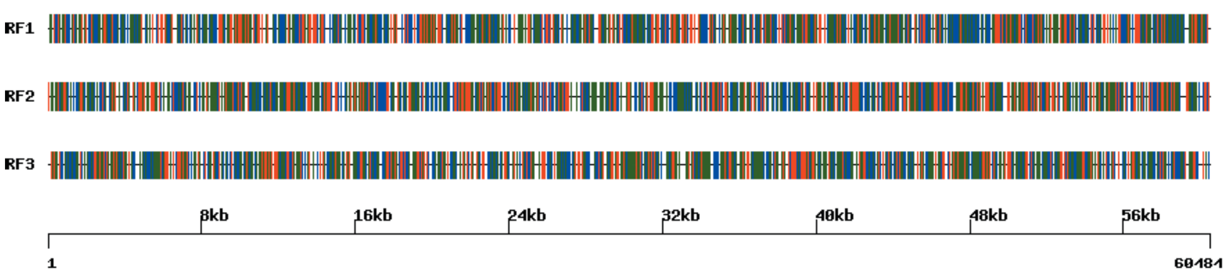

Exon ORF (Before Splicing)

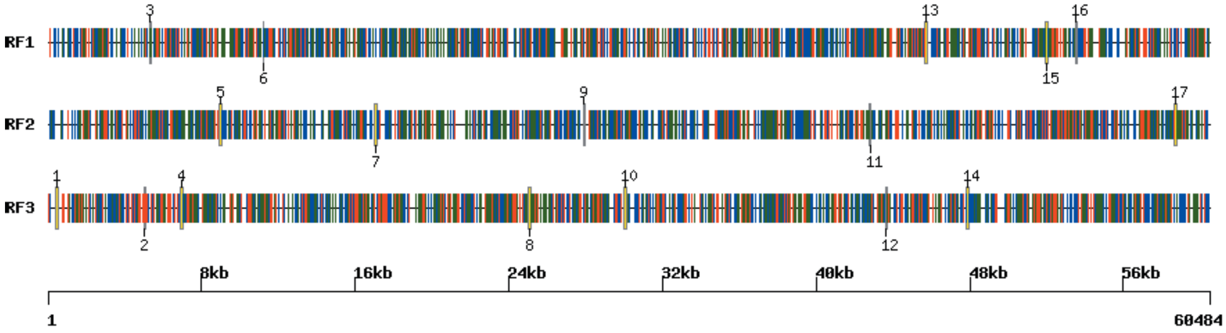

Exon ORF (After Splicing)

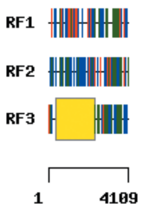

GTT : | ACA : | CTG : |

Exon ARF (Before Splicing)

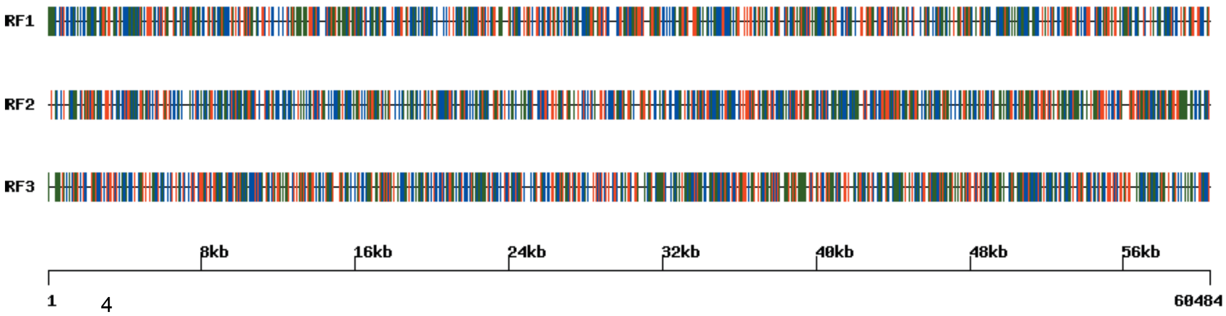

Exon ARF (After Splicing)

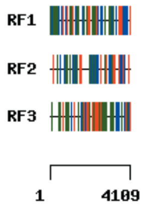

Exon ORF Plot for Gene CARD10

[Genome: Homo sapiens] [Gene Name: caspase recruitment domain protein 10]

Gene ID: **29775** Gene Symbol: CARD10 Gene Length: 28811 Number of Exons: 20

**Exon**	1	2	3	4	5	6	7	8	9	10	11	12	13	14	15	16	17	18	19	20
**Length**	235	138	326	210	156	126	192	135	116	29	124	160	112	244	70	103	159	116	113	235

Total Spliced Exon Length: 3099

TAA: | TGA: | TAG: | Exon:

Random Sequence ORF

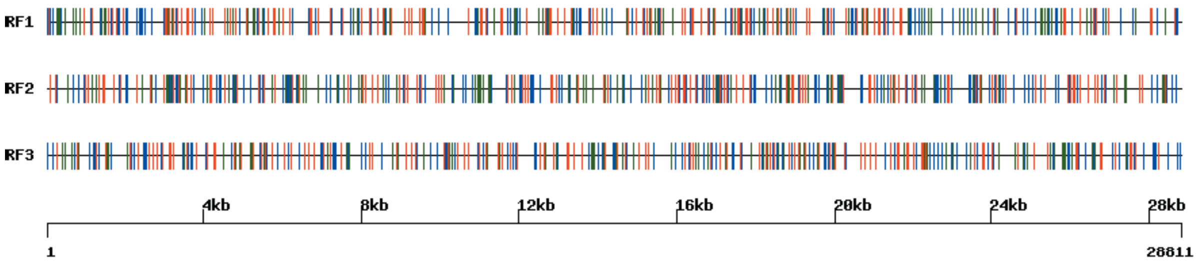

Exon ORF (Before Splicing)

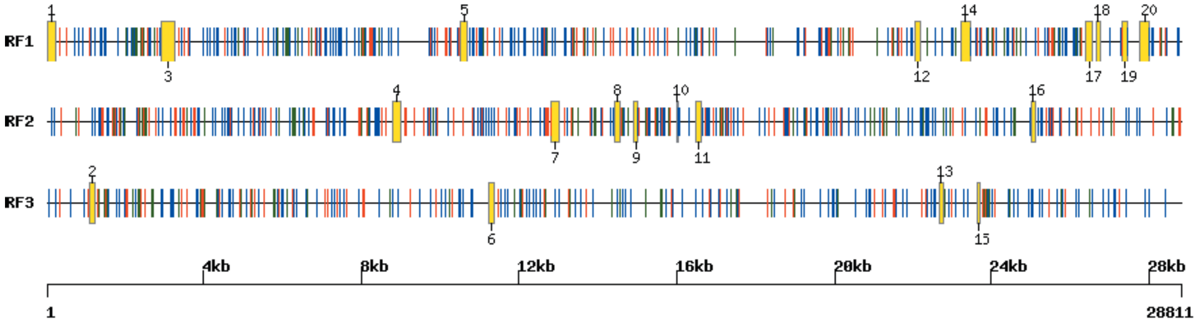

Exon ORF (After Splicing)

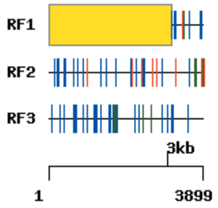

GTT : | ACA : | CTG : |

Exon ARF (Before Splicing)

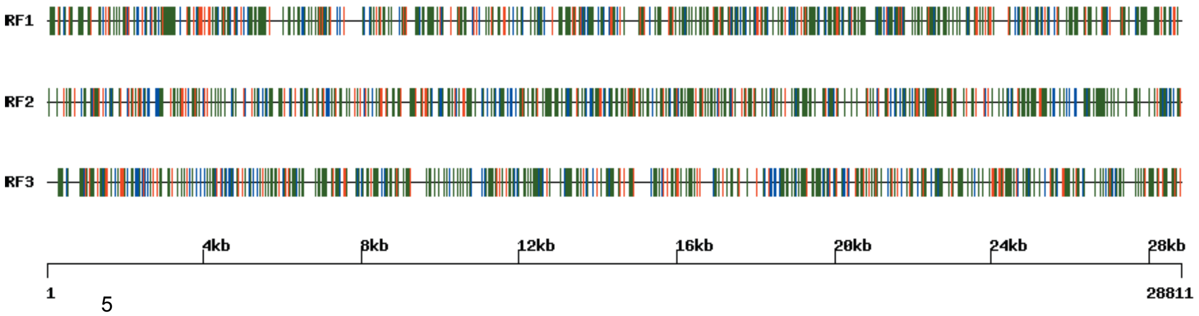

Exon ARF (After Splicing)

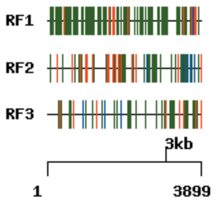

Exon ORF Plot for Gene HADHA

[Genome: Homo sapiens] [Gene Name: hydroxyacyl dehydrogenase, subunit A]

Gene ID: **3030** Gene Symbol: HADHA Gene Length: 53981 Number of Exons: 20

**Exon**	1	2	3	4	5	6	7	8	9	10	11	12	13	14	15	16	17	18	19	20
**Length**	67	42	71	134	139	120	103	123	119	57	110	135	172	87	141	69	196	115	146	146

Total Spliced Exon Length: 2292

TAA: | TGA: | TAG: | Exon:

Random Sequence ORF

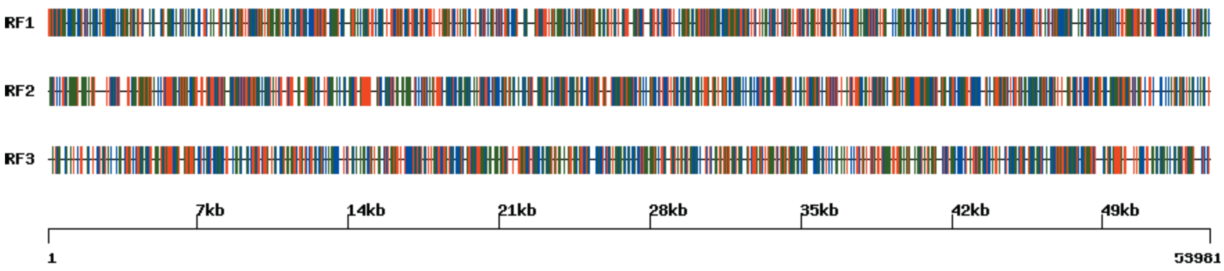

Exon ORF (Before Splicing)

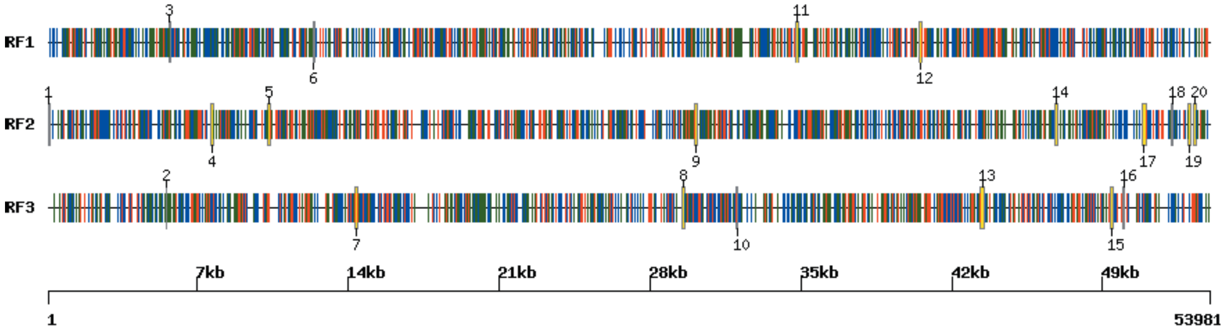

Exon ORF (After Splicing)

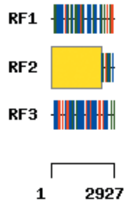

GTT : | ACA : | CTG : |

Exon ARF (Before Splicing)

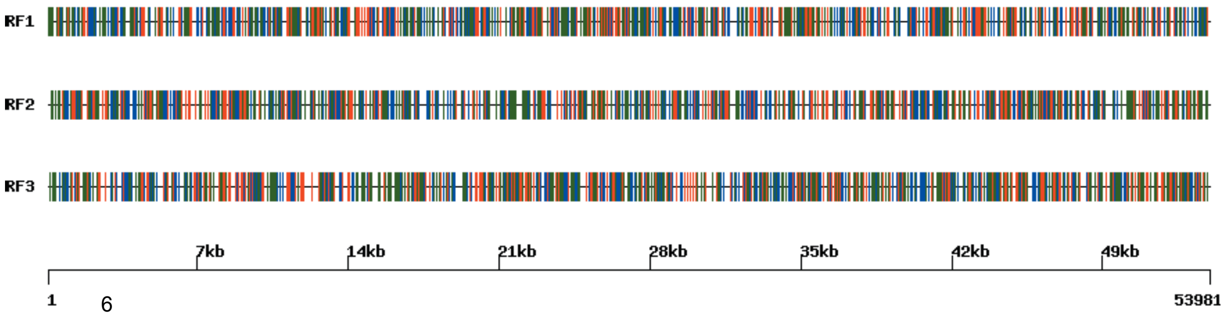

Exon ARF (After Splicing)

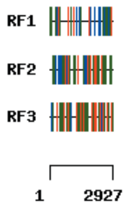

Exon ORF Plot for Gene HK2

[Genome: Homo sapiens] [Gene Name: hexokinase 2]

Gene ID: **3099** Gene Symbol: HK2 Gene Length: 60700 Number of Exons: 18

**Exon**	1	2	3	4	5	6	7	8	9	10	11	12	13	14	15	16	17	18
**Length**	63	163	149	120	96	100	184	156	234	305	149	120	96	100	184	156	234	145

Total Spliced Exon Length: 2754

TAA: | TGA: | TAG: | Exon:

Random Sequence ORF

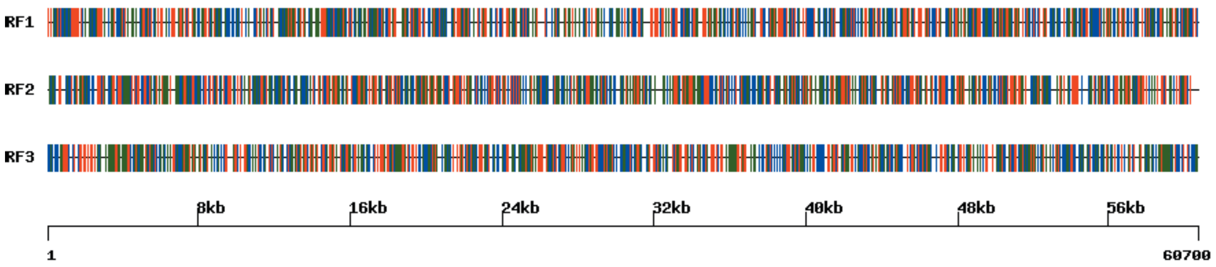

Exon ORF (Before Splicing)

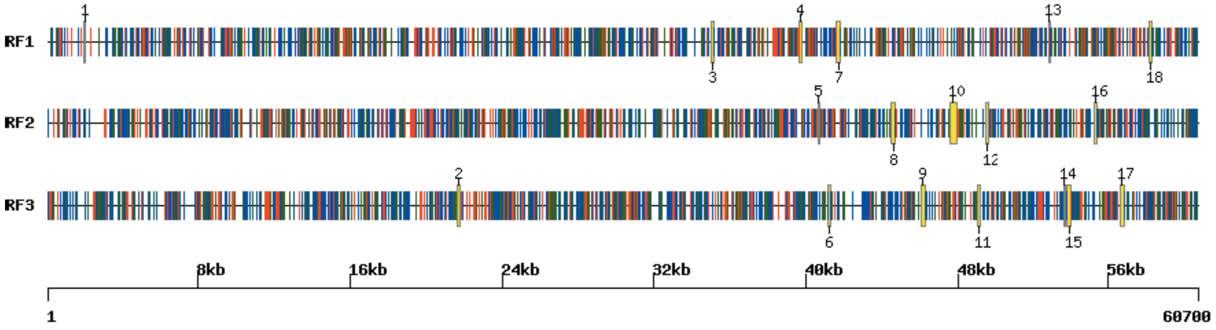

Exon ORF (After Splicing)

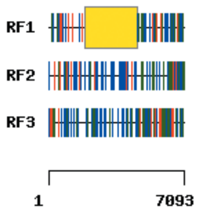

GTT : | ACA : | CTG : |

Exon ARF (Before Splicing)

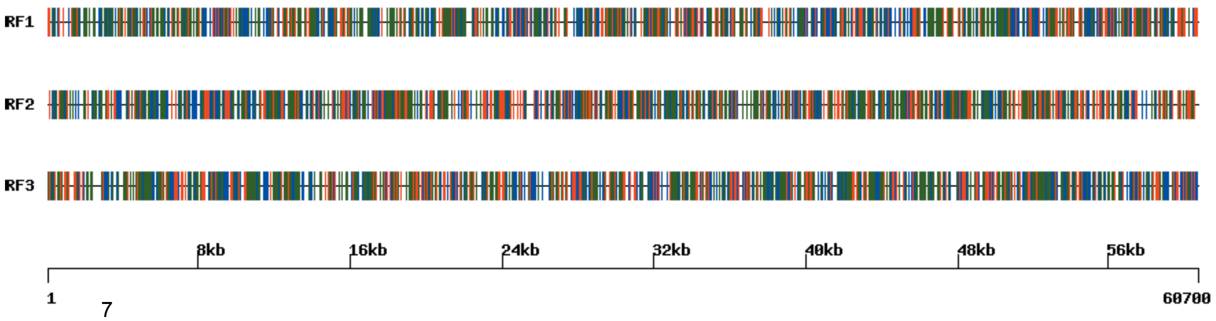

Exon ARF (After Splicing)

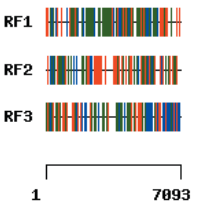

Exon ORF Plot for Gene CIITA

[Genome: Homo sapiens] [Gene Name: class II transactivator]

Gene ID: **4261** Gene Symbol: CIITA Gene Length: 47784 Number of Exons: 19

**Exon**	1	2	3	4	5	6	7	8	9	10	11	12	13	14	15	16	17	18	19
**Length**	52	147	96	63	78	45	147	144	165	69	1651	159	72	81	93	87	84	84	76

Total Spliced Exon Length: 3393

TAA: | TGA: | TAG: | Exon:

Random Sequence ORF

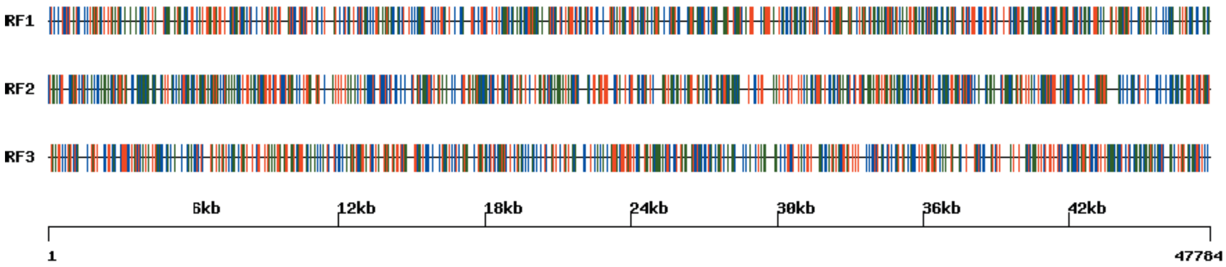

Exon ORF (Before Splicing)

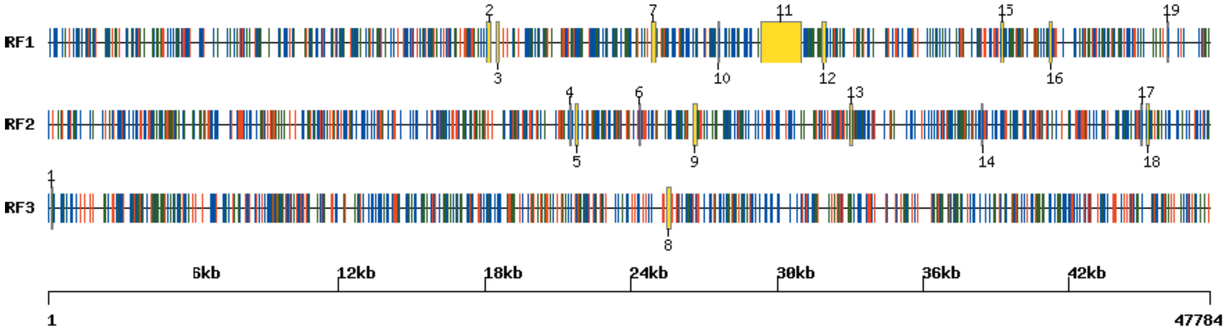

Exon ORF (After Splicing)

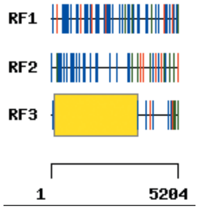

GTT : | ACA : | CTG : |

Exon ARF (Before Splicing)

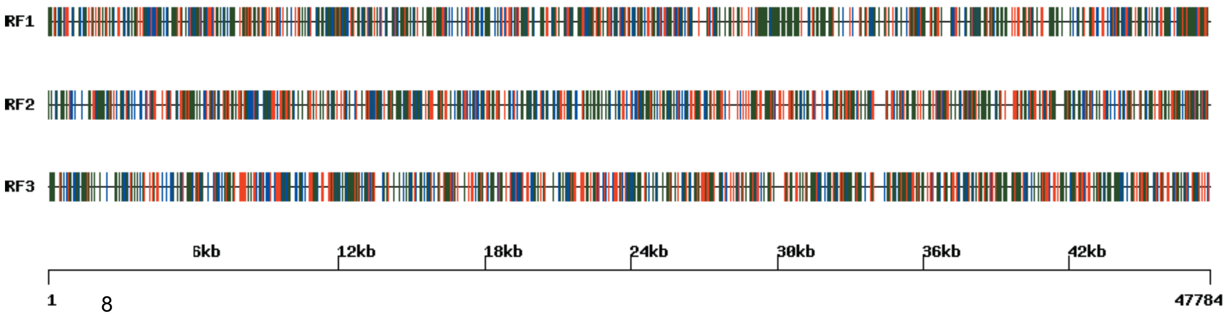

Exon ARF (After Splicing)

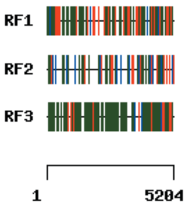

Exon ORF Plot for Gene ABCB10

[Genome: Homo sapiens] [Gene Name: ATP-binding cassette, sub-family B, member 10]

Gene ID: **23456** Gene Symbol: ABCB10 Gene Length: 42113 Number of Exons: 13

**Exon**	1	2	3	4	5	6	7	8	9	10	11	12	13
**Length**	517	201	203	135	147	136	96	210	80	181	44	35	232

Total Spliced Exon Length: 2217

TAA: | TGA: | TAG: | Exon:

Random Sequence ORF

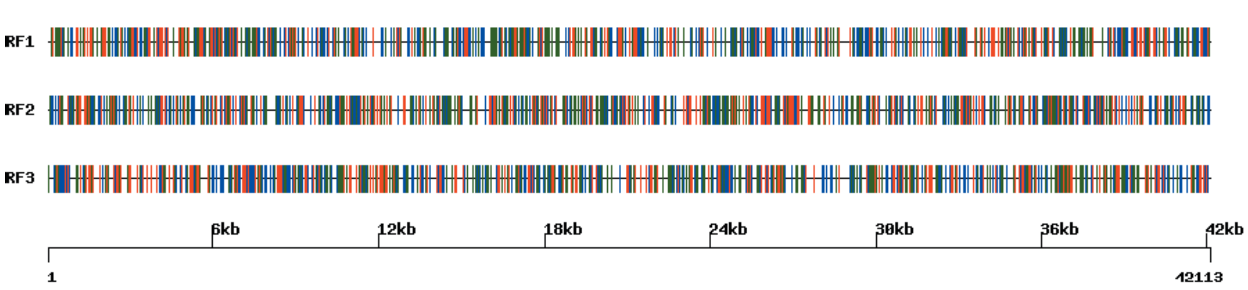

Exon ORF (Before Splicing)

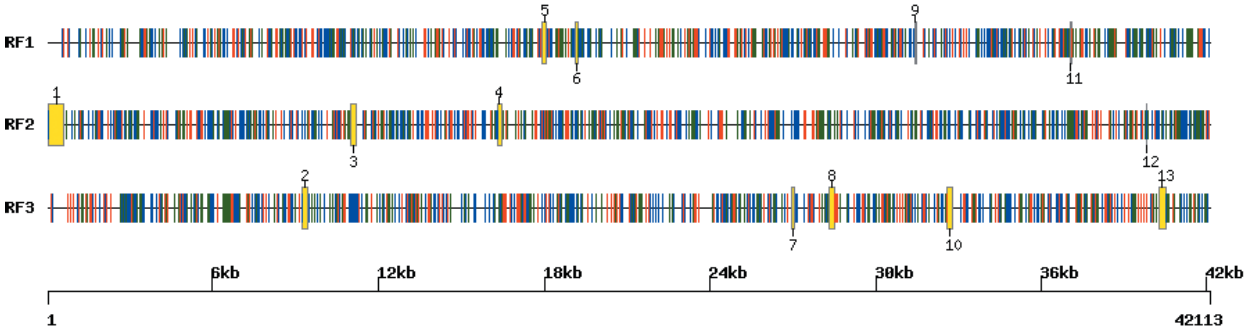

Exon ORF (After Splicing)

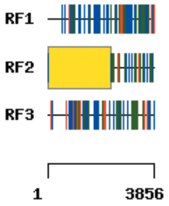

GTT : | ACA : | CTG : |

Exon ARF (Before Splicing)

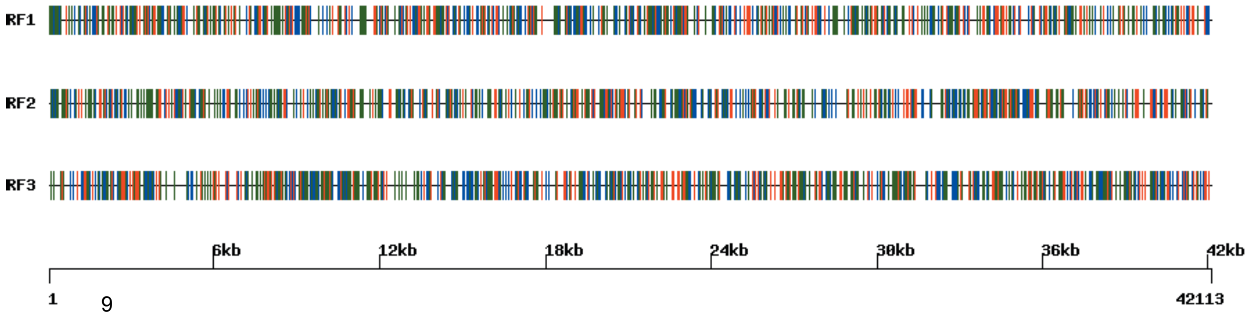

Exon ARF (After Splicing)

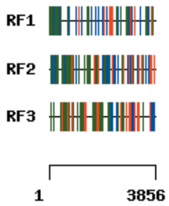

Exon ORF Plot for Gene API5

[Genome: Homo sapiens] [Gene Name: apoptosis inhibitor 5]

Gene ID: **8539** Gene Symbol: API5 Gene Length: 32547 Number of Exons: 14

**Exon**	1	2	3	4	5	6	7	8	9	10	11	12	13	14
**Length**	69	162	94	66	152	207	105	90	182	94	57	77	137	23

Total Spliced Exon Length: 1515

TAA: | TGA: | TAG: | Exon:

Random Sequence ORF

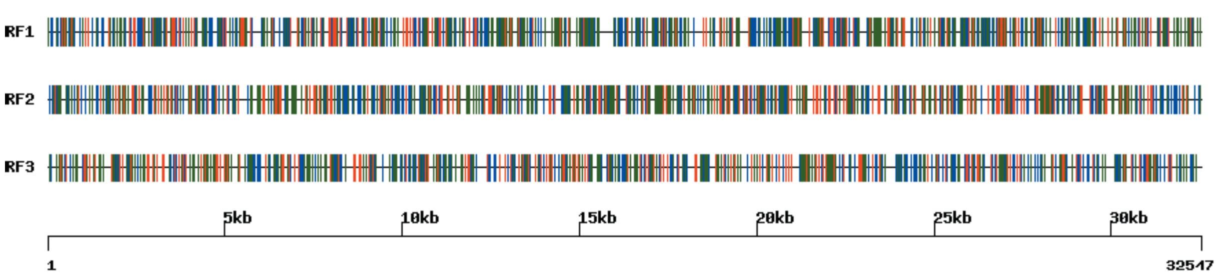

Exon ORF (Before Splicing)

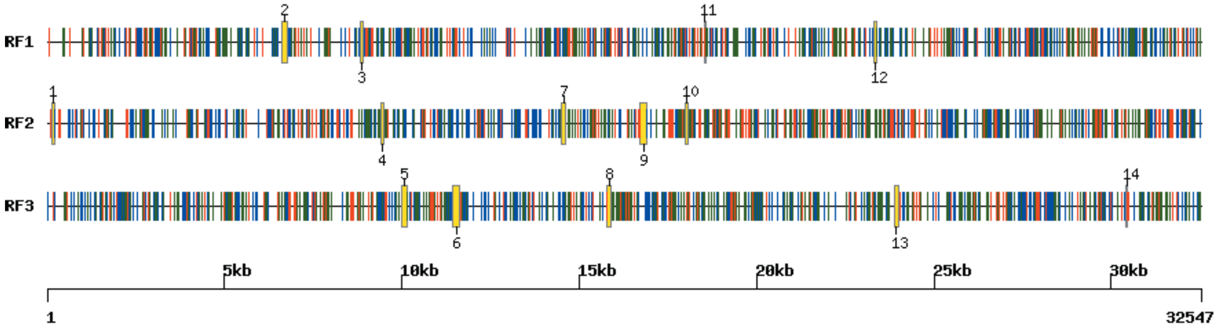

Exon ORF (After Splicing)

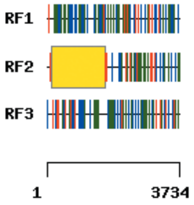

GTT : | ACA : | CTG : |

Exon ARF (Before Splicing)

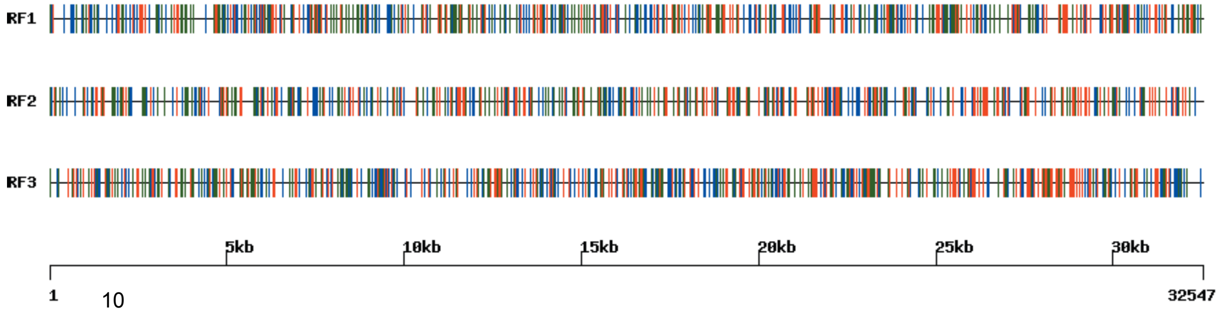

Exon ARF (After Splicing)

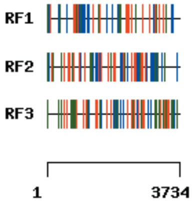

Exon ORF Plot for Gene CTPS

[Genome: Homo sapiens] [Gene Name: CTP synthase]

Gene ID: **1503** Gene Symbol: CTPS Gene Length: 32786 Number of Exons: 17

**Exon**	1	2	3	4	5	6	7	8	9	10	11	12	13	14	15	16	17
**Length**	166	171	101	117	84	81	152	133	89	95	63	44	97	56	97	145	85

Total Spliced Exon Length: 1776

TAA: | TGA: | TAG: | Exon:

Random Sequence ORF

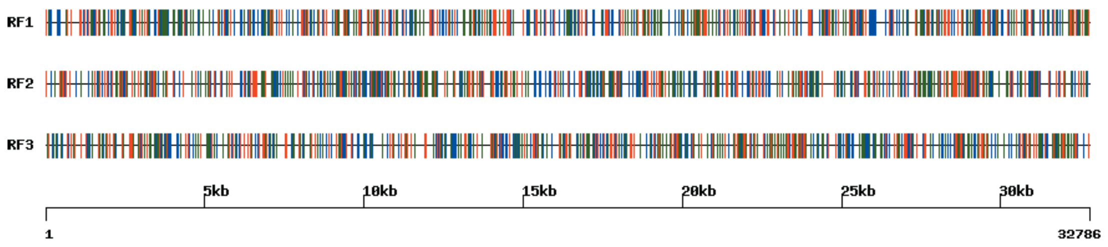

Exon ORF (Before Splicing)

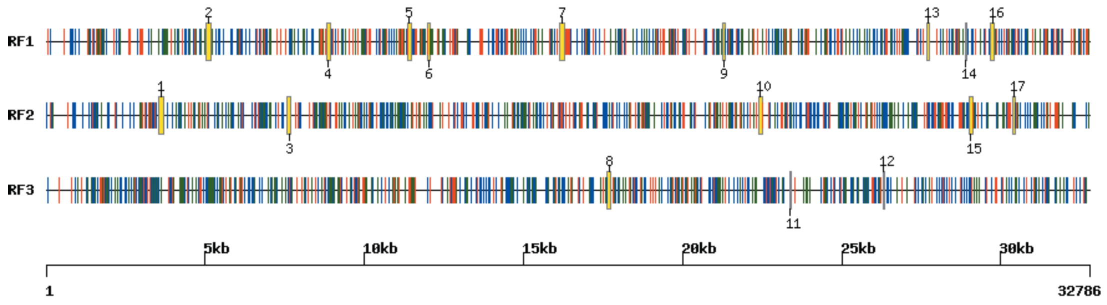

Exon ORF (After Splicing)

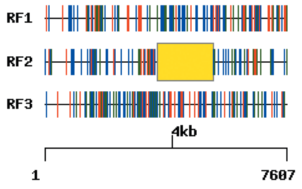

GTT : | ACA : | CTG : |

Exon ARF (Before Splicing)

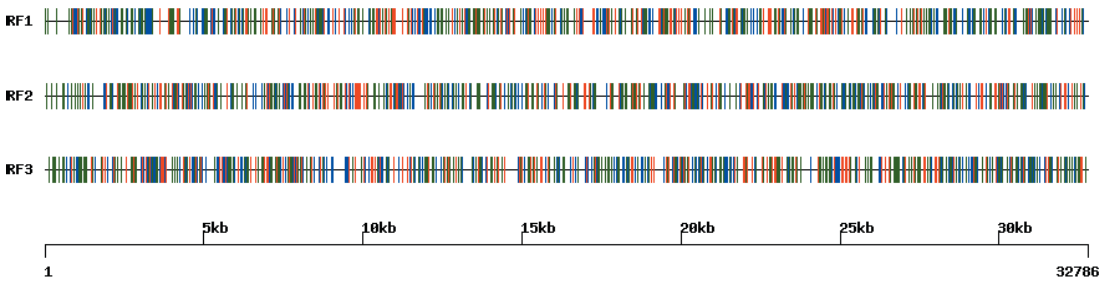

Exon ARF (After Splicing)

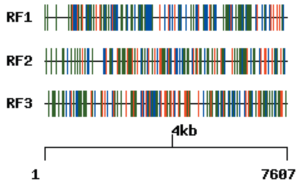

Exon ORF Plot for Gene LIPF

[Genome: Homo sapiens] [Gene Name: lipase, gastric]

Gene ID: **8513** | Gene Symbol: LIPF | Gene Length: 14347 | Number of Exons: 9

**Exon**	1	2	3	4	5	6	7	8	9
**Length**	105	118	199	110	137	147	72	72	237

Total Spliced Exon Length: 1197

TAA: | TGA: | TAG: | Exon:

Random Sequence ORF

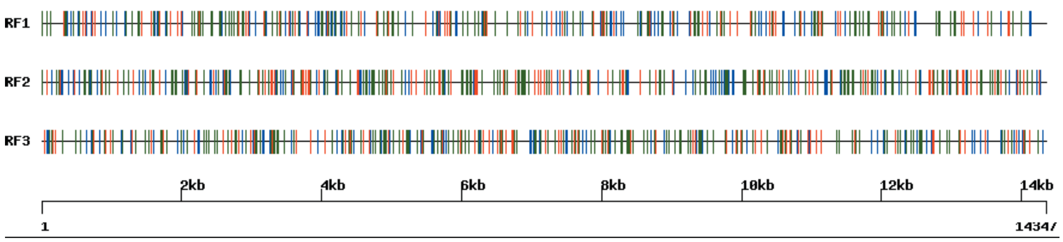

Exon ORF (Before Splicing)

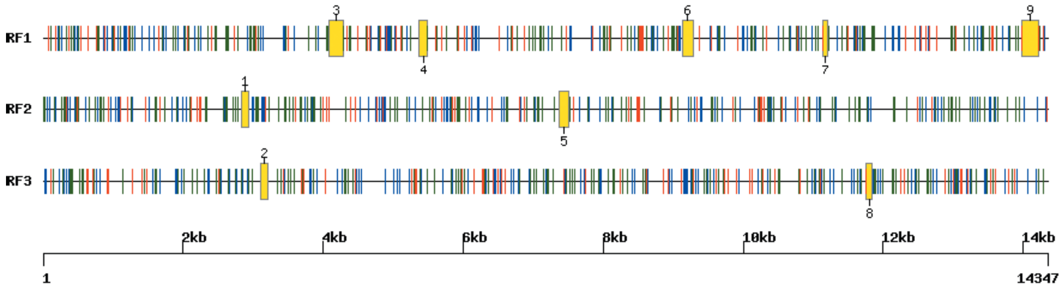

Exon ORF (After Splicing)

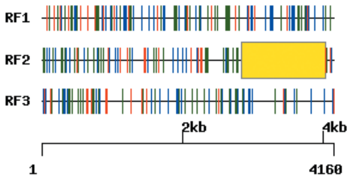

GTT : | ACA : | CTG : |

Exon ARF (Before Splicing)

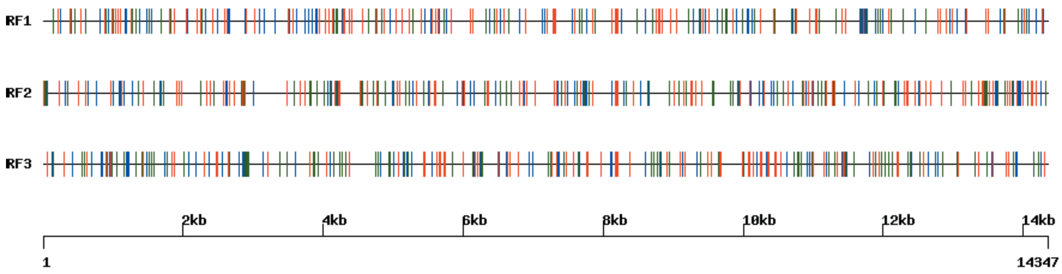

Exon ARF (After Splicing)

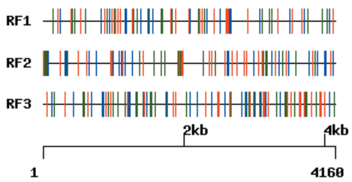

Exon ORF Plot for Gene IFI44

[Genome: Homo sapiens] [Gene Name: interferon-induced, hepatitis C-associated microtubular aggregat]

Gene ID: **10561** Gene Symbol: IFI44 Gene Length: 14242 Number of Exons: 8

**Exon**	1	2	3	4	5	6	7	8
**Length**	457	37	196	150	172	101	175	47

Total Spliced Exon Length: 1335

TAA: | TGA: | TAG: | Exon:

Random Sequence ORF

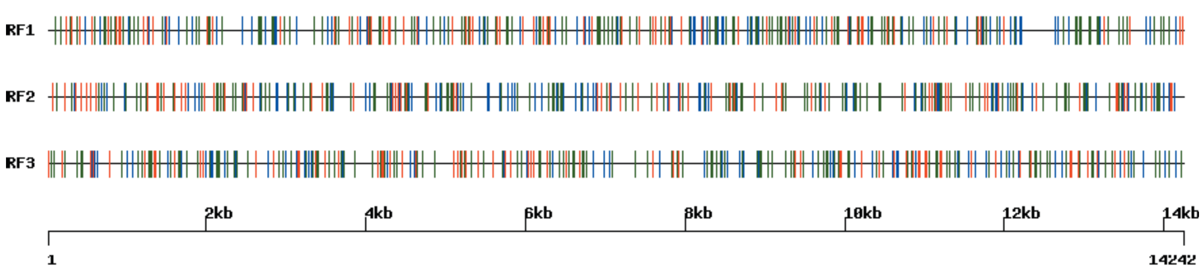

Exon ORF (Before Splicing)

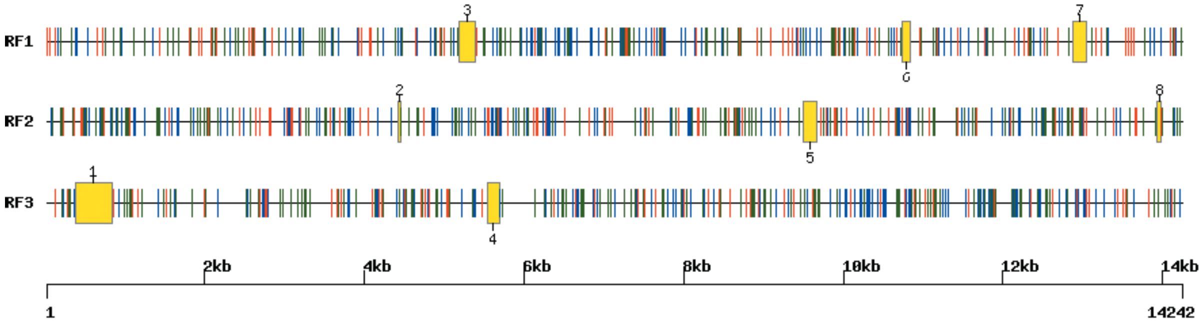

Exon ORF (After Splicing)

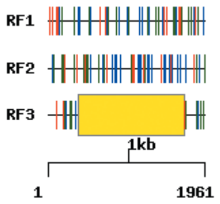

GTT : | ACA : | CTG : |

Exon ARF (Before Splicing)

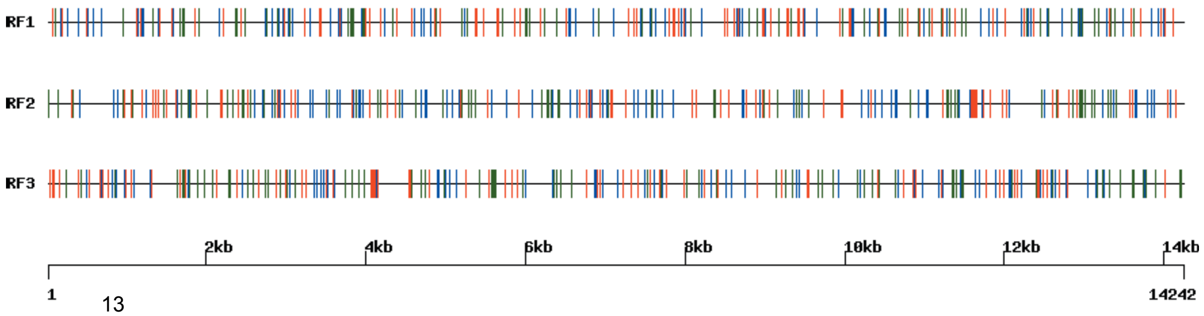

Exon ARF (After Splicing)

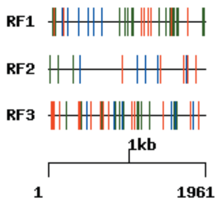

Exon ORF Plot for Gene GBA

[Genome: Homo sapiens] [Gene Name: glucocerebrosidase precursor]

Gene ID: **2629** Gene Symbol: GBA Gene Length: 10246 Number of Exons: 11

**Exon**	1	2	3	4	5	6	7	8	9	10	11
**Length**	27	88	192	147	134	173	238	225	164	117	106

Total Spliced Exon Length: 1611

TAA: | TGA: | TAG: | Exon:

Random Sequence ORF

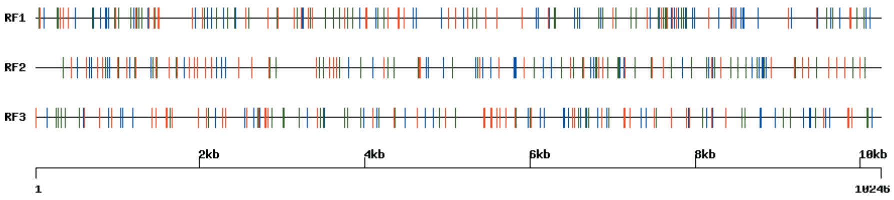

Exon ORF (Before Splicing)

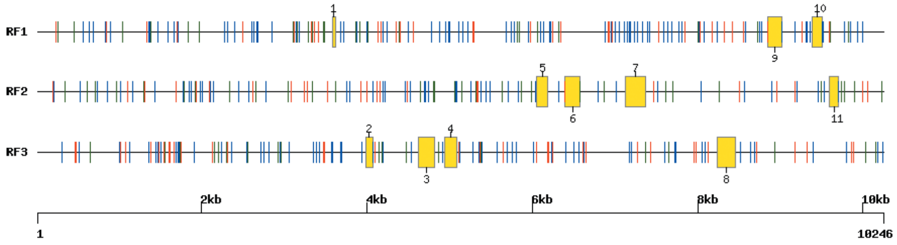

Exon ORF (After Splicing)

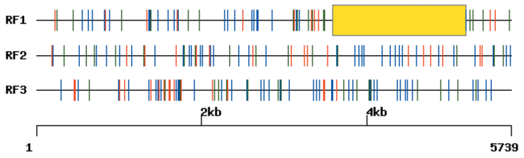

GTT : | ACA : | CTG : |

Exon ARF (Before Splicing)

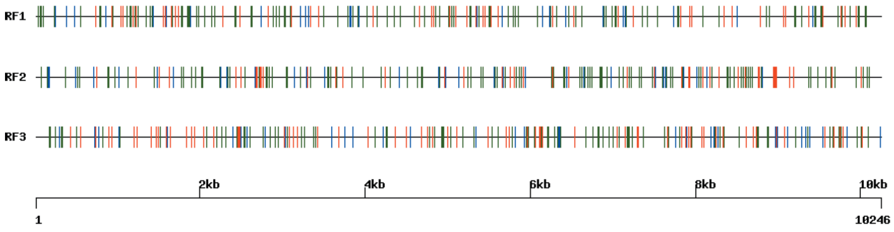

Exon ARF (After Splicing)

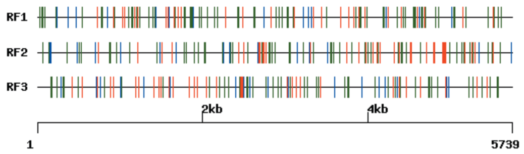

Representative invertebrate (*C. elegans*) genes

EOPs of three representative *C. elegans* genes are displayed on pp. 16-18. *C. elegans* gene sequences are also very similar to random DNA with 99.95% of their ORFs being shorter than 750 bases. The ARFs in *C. elegans* genes also exhibit random characteristics. The vast majority of *C. elegans* exons (98%) are shorter than 750 bases, and >90% are shorter than 400 bases. Very rarely did the *C. elegans* exons not conform to the random sequence model. *C. elegans* genes are fairly intron dense, having an average of 5 introns per gene.

Exon ORF Plot for Gene K08F4.1

[Genome: Caenorhabditis elegans] [Gene Name:]

Gene ID: **177879** Gene Symbol: K08F4.1 Gene Length: 4525 Number of Exons: 11

**Exon**	1	2	3	4	5	6	7	8	9	10	11
**Length**	65	167	239	203	169	458	253	264	264	216	255

Total Spliced Exon Length: 2553

TAA: | TGA: | TAG: | Exon:

Random Sequence ORF

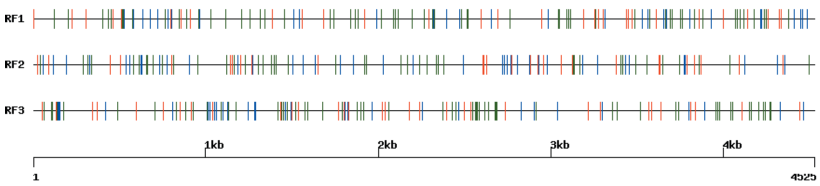

Exon ORF (Before Splicing)

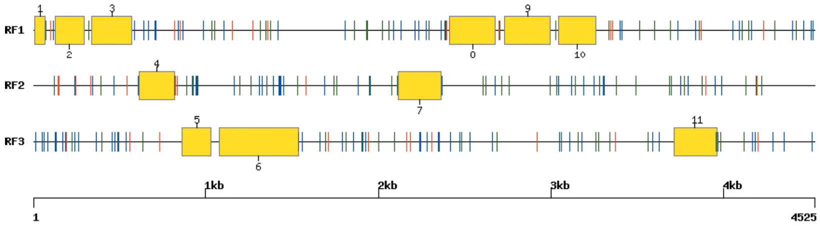

Exon ORF (After Splicing)

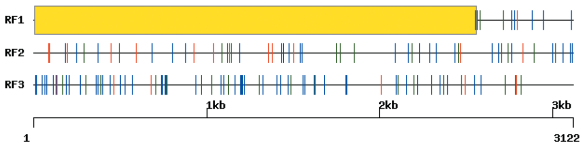

GTT : | ACA : | CTG : |

Exon ARF (Before Splicing)

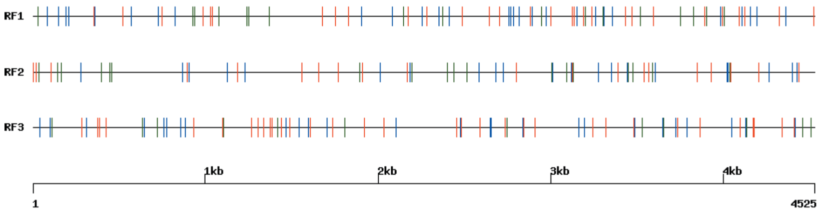

Exon ARF (After Splicing)

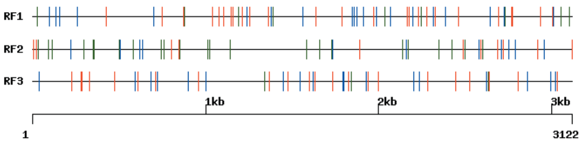

Exon ORF Plot for Gene C34G6.1

[Genome: Caenorhabditis elegans] [Gene Name:]

Gene ID: **172265** Gene Symbol: C34G6.1 Gene Length: 11071 Number of Exons: 15

**Exon**	1	2	3	4	5	6	7	8	9	10	11	12	13	14	15
**Length**	34	152	313	82	220	525	376	192	814	103	171	117	686	1264	258

Total Spliced Exon Length: 5307

TAA: | TGA: | TAG: | Exon:

Random Sequence ORF

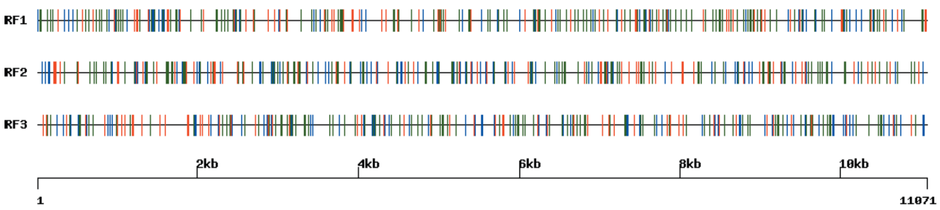

Exon ORF (Before Splicing)

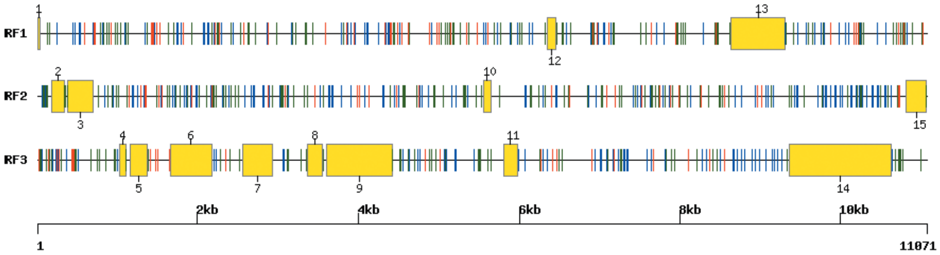

Exon ORF (After Splicing)

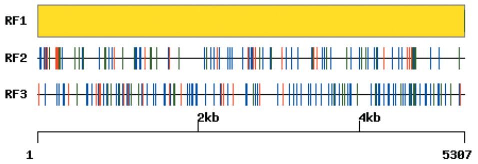

GTT : | ACA : | CTG : |

Exon ARF (Before Splicing)

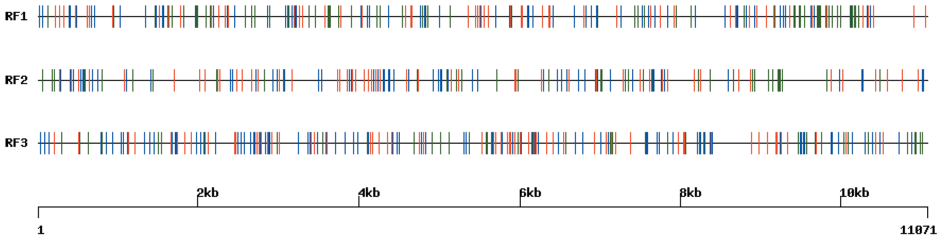

Exon ARF (After Splicing)

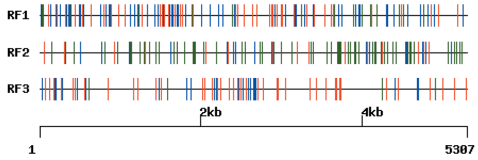

Exon ORF Plot for Gene F23F12.13

[Genome: Caenorhabditis elegans] [Gene Name:]

Gene ID: **3896740** Gene Symbol: F23F12.13 Gene Length: 2497 Number of Exons: 9

**Exon**	1	2	3	4	5	6	7	8	9
**Length**	90	80	254	146	131	319	157	102	308

Total Spliced Exon Length: 1587

TAA: | TGA: | TAG: | Exon:

Random Sequence ORF

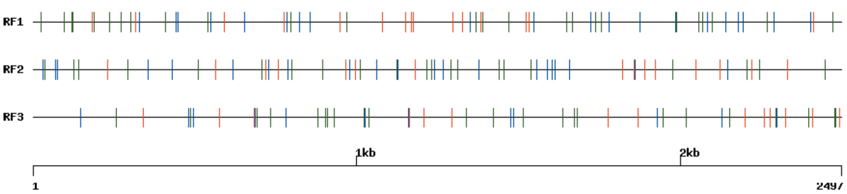

Exon ORF (Before Splicing)

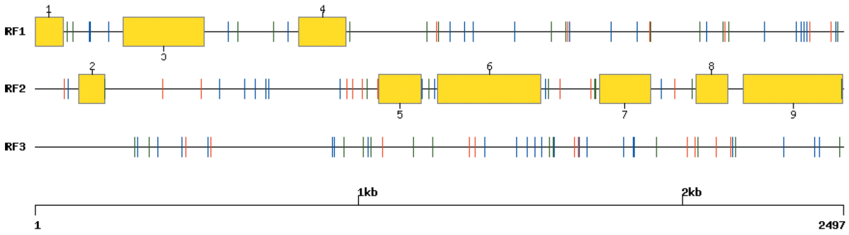

Exon ORF (After Splicing)

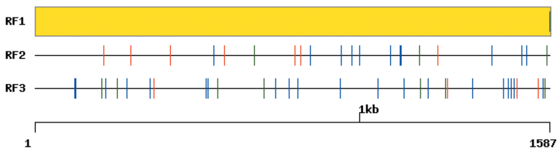

GTT : | ACA : | CTG : |

Exon ARF (Before Splicing)

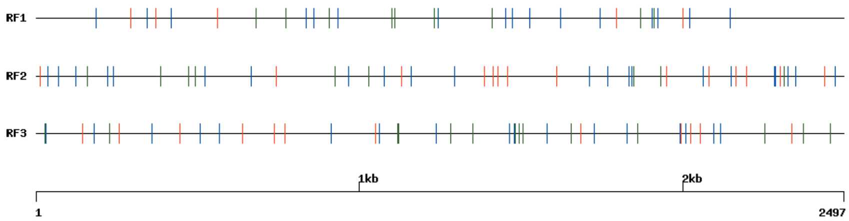

Exon ARF (After Splicing)

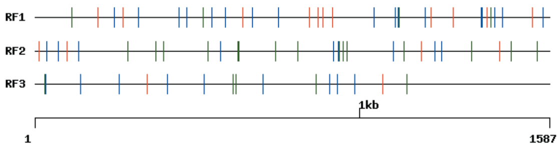

Representative parasitic protist (*P. falciparum*) genes

EOPs of two representative *P. falciparum* genes are displayed on pp. 20-21. *P. falciparum* gene sequences are non-random only with respect to the lengths of a small subset of ORFs that specifically contain the non-conforming exons of genes. The ARFs in *P. falciparum* genes exhibit random characteristics. Nearly all, 99.87%, of the ORFs in *P. falciparum* genomes are shorter than 750 bases, and 69% of exons are shorter than 750 bases. The 31% non-conforming *P. falciparum* exons occur conspicuously within the rarely occurring non-conforming ORFs against a background of random ORFs, indicating that these must have been derived from splicing of short exons. *P. falciparum* genes have a relatively low density of introns (with average of 1 intron per gene) compared to *H. sapiens*, *A. thaliana* and *C. elegans*.

Exon ORF Plot for Gene PFB0365w

[Genome: Plasmodium falciparum] [Gene Name: hypothetical protein]

Gene ID: **812673** Gene Symbol: PFB0365w Gene Length: 7272 Number of Exons: 16

**Exon**	1	2	3	4	5	6	7	8	9	10	11	12	13	14	15	16
**Length**	90	37	92	124	69	74	62	73	110	263	92	43	235	78	70	3894

Total Spliced Exon Length: 5406

TAA: | TGA: | TAG: | Exon:

Random Sequence ORF

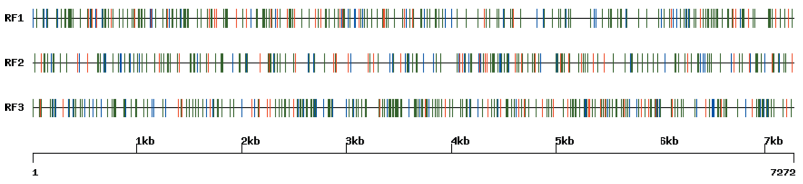

Exon ORF (Before Splicing)

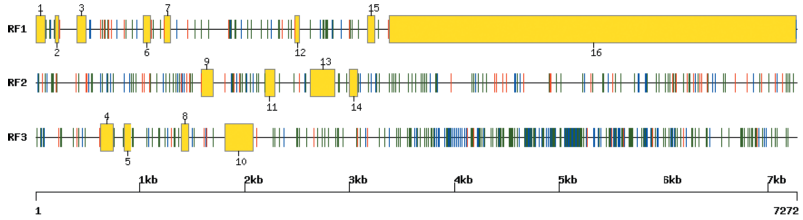

Exon ORF (After Splicing)

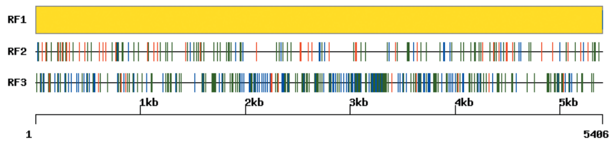

GTT : | ACA : | CTG : |

Exon ARF (Before Splicing)

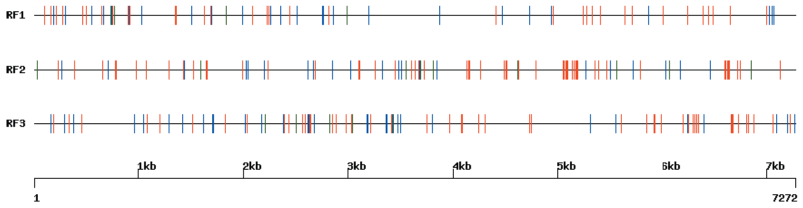

Exon ARF (After Splicing)

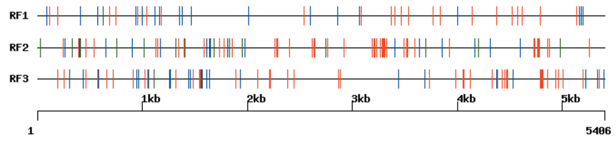

Exon ORF Plot for Gene PFC0990c

[Genome: Plasmodium falciparum]			[Gene Name: hypothetical protein]
Gene ID: **814536**	Gene Symbol: PFC0990c	Gene Length: 3819	Number of Exons: 14

**Exon**	1	2	3	4	5	6	7	8	9	10	11	12	13	14
**Length**	46	1307	42	90	86	82	65	63	152	47	35	55	58	134

Total Spliced Exon Length: 2262

TAA: | TGA: | TAG: | Exon:

Random Sequence ORF

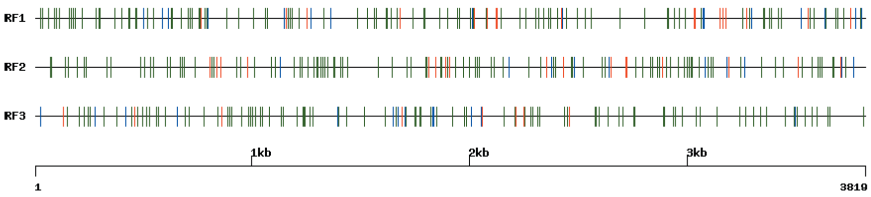

Exon ORF (Before Splicing)

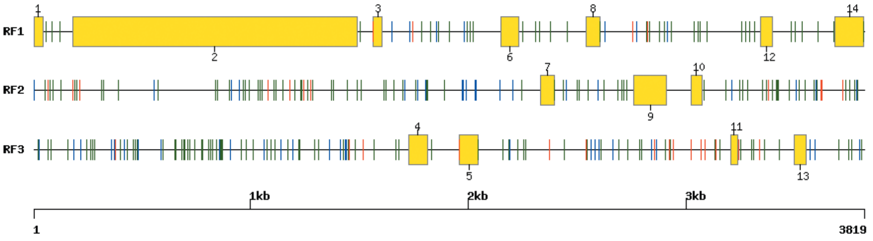

Exon ORF (After Splicing)

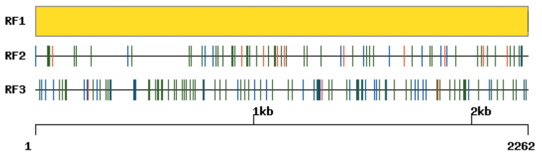

GTT : | ACA : | CTG : |

Exon ARF (Before Splicing)

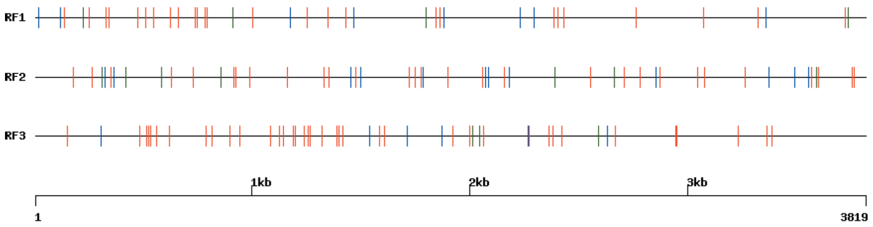

Exon ARF (After Splicing)

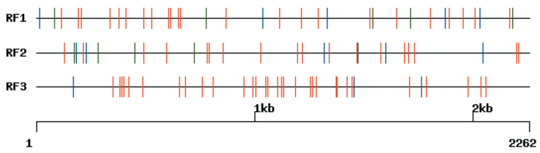

Representative yeast (*S. pombe*) genes

EOPs of two representative *S. pombe* genes are displayed on pp. 23-24. *S. pombe* gene sequences are non-random only with respect to the lengths of the small subset of ORFs that specifically contain the non-conforming exons. The ARFs in *S. pombe* genes do exhibit random characteristics. Nearly all, 99.77%, of the ORFs in *S. pombe* genomes are shorter than 750 bases, and 67% of their exons are shorter than 750 bases. The remaining 33% of non-conforming *S. pombe* exons occur conspicuously within the rarely occurring non-conforming ORFs against a background of random ORFs, indicating that these must have been derived from splicing of short exons. Of the eukaryote genomes we examined, *S. pombe* genes had the lowest density of introns (with an average of 1 intron per gene).

Exon ORF Plot for Gene cdc5

[Genome: Schizosaccharomyces pombe] [Gene Name: hypothetical protein]

Gene ID: **2543583** Gene Symbol: cdc5 Gene Length: 2577 Number of Exons: 6

**Exon**	1	2	3	4	5	6
**Length**	19	20	15	120	449	1651

Total Spliced Exon Length: 2274

TAA: | TGA: | TAG: | Exon:

Random Sequence ORF

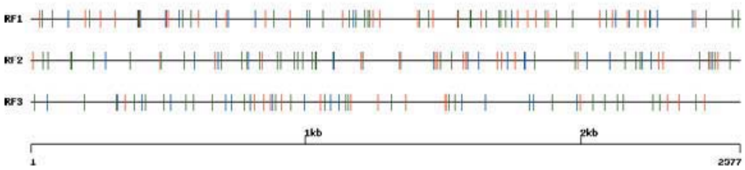

Exon ORF (Before Splicing)

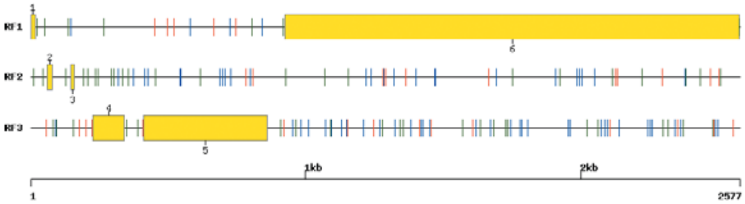

Exon ORF (After Splicing)

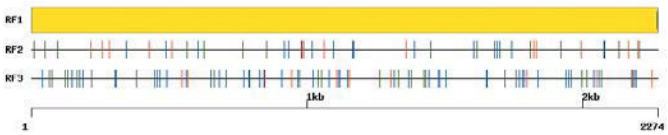

GTT : | ACA : | CTG : |

Exon ARF (Before Splicing)

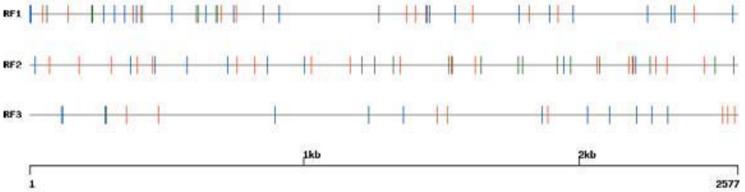

Exon ARF (After Splicing)

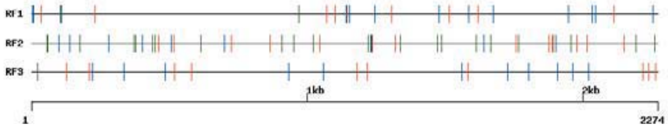

Exon ORF Plot for Gene cki2

[Genome: Schizosaccharomyces pombe] [Gene Name: serine/threonine protein kinase]

Gene ID: **2541341** Gene Symbol: cki2 Gene Length: 1492 Number of Exons: 4

**Exon**	1	2	3	4
**Length**	126	70	149	963

Total Spliced Exon Length: 1308

TAA: | TGA: | TAG: | Exon:

Random Sequence ORF

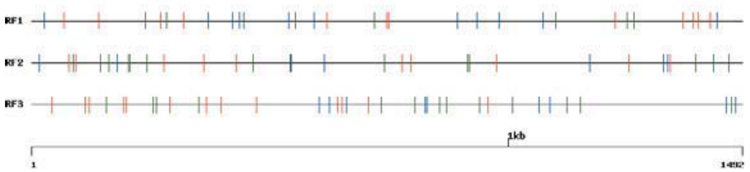

Exon ORF (Before Splicing)

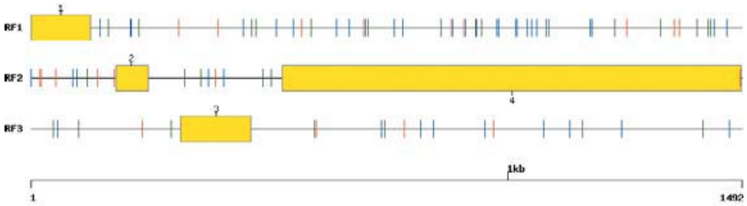

Exon ORF (After Splicing)

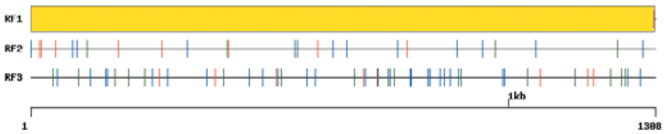

GTT : | ACA : | CTG : |

Exon ARF (Before Splicing)

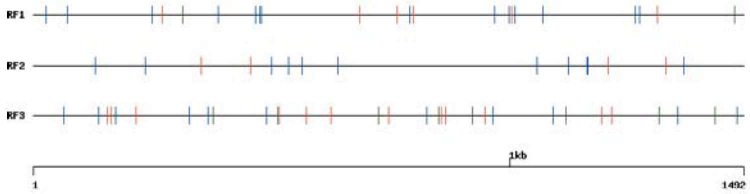

Exon ARF (After Splicing)

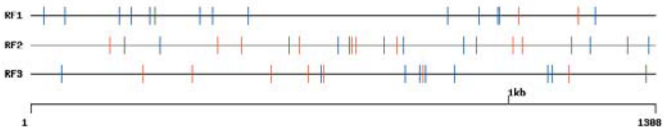

Representative bacterial (*E. coli*) genes

EOPs of two representative *E. coli* genes are displayed on pp. 26-27. E.*coli* gene sequences are non-random only with respect to the lengths of the small subset of ORFs that specifically contain the non-conforming coding sequences of their genes. The ARFs in *E. coli* genes exhibit clearly random characteristics. We found that 99.2% of the ORFs in the *E. coli* genome are shorter than 750 bases, and that 43% of their coding sequences are shorter than 750 bases. Distinct from the eukaryotes, a majority of the *E. coli* coding sequences (57%) did not conform to the random sequence model. All of the non-conforming coding sequences in the *E. coli* genome were contained within the 0.8% non-conforming ORFs. *E. coli* genes lack introns altogether. Consistent with these observations, the *E. coli* genome is the smallest of those examined, and contains only contiguously coding genes without introns or intergenic sequences.

Exon ORF Plot for Gene ackA

[Genome: Escherichia coli K12] [Gene Name: acetate/propionate kinase]

Gene ID: **946775** Gene Symbol: ackA Gene Length: 1203

TAA: | TGA: | TAG: | Exon:

Random Sequence ORF

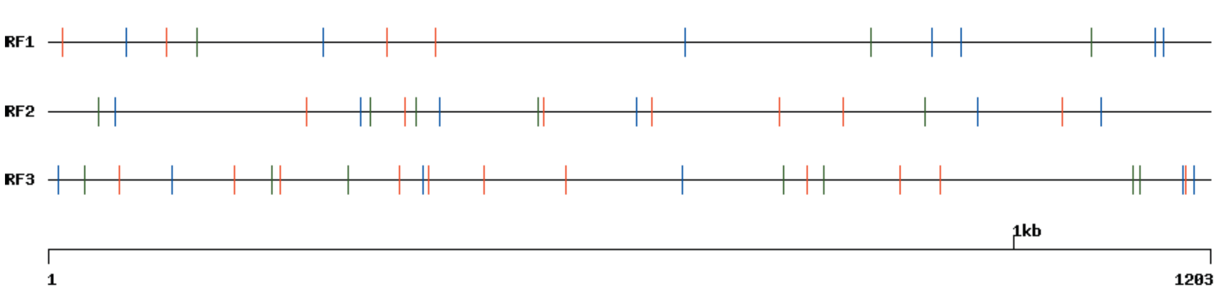

Gene ORF

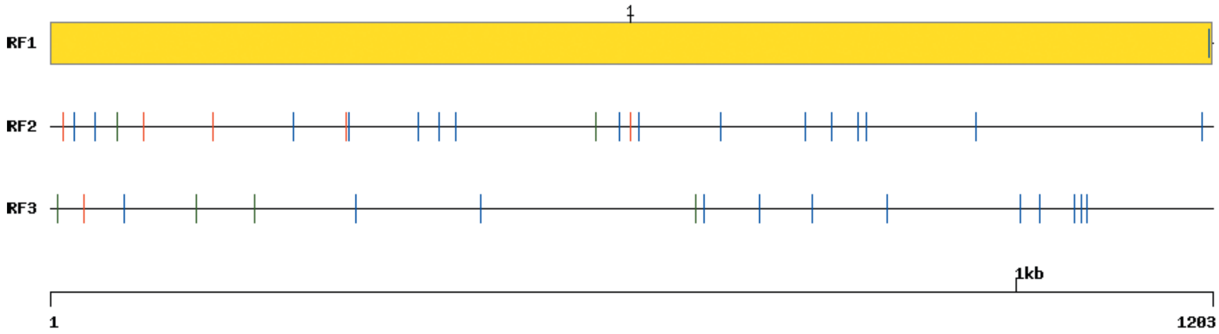

GTT : | ACA : | CTG : |

Gene ARF

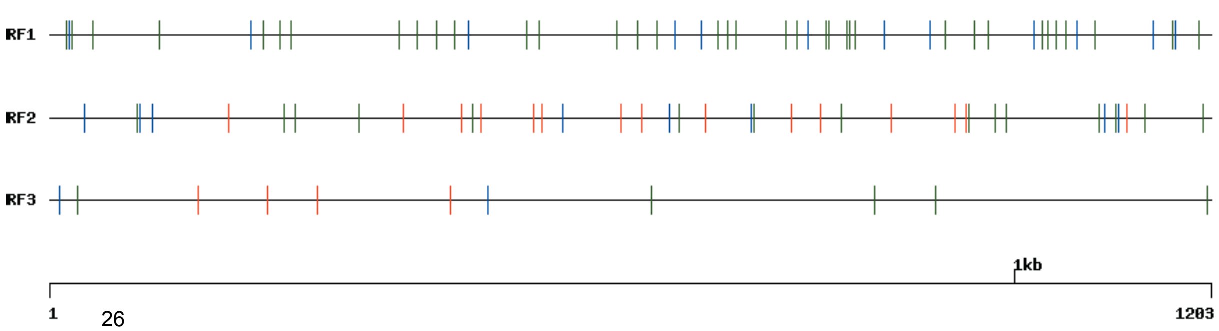

Exon ORF Plot for Gene acrB

[Genome: Escherichia coli K12] [Gene Name: multidrug efflux system protein]

Gene ID: **945108** Gene Symbol: acrB Gene Length: 3150

TAA: | TGA: | TAG: | Exon:

Random Sequence ORF

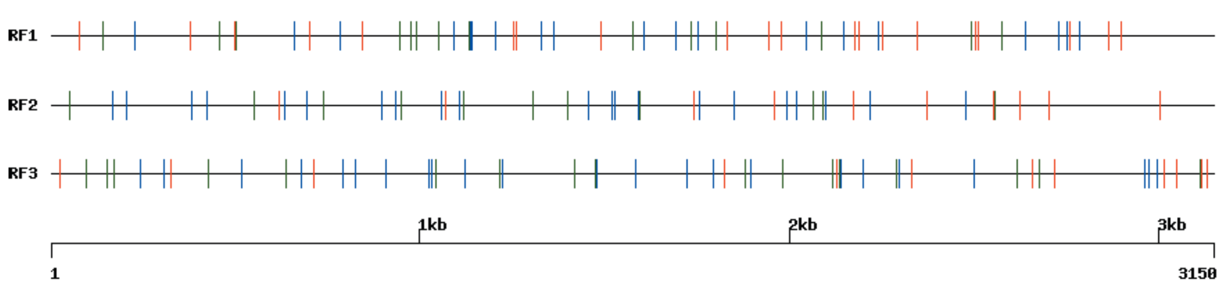

Gene ORF

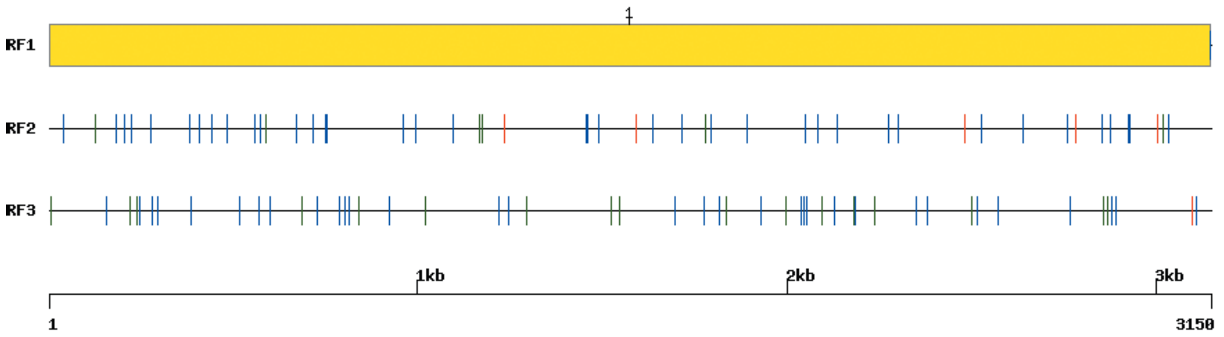

GTT : | ACA : | CTG : |

Gene ARF

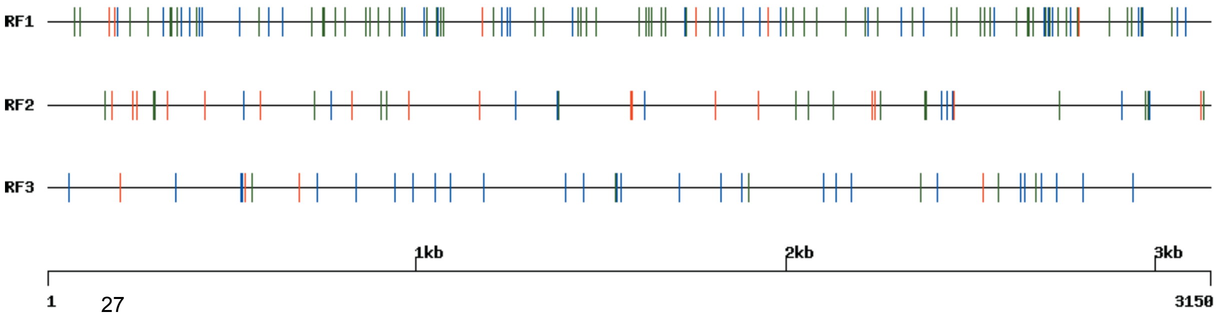

Supplement: Figure S1 — (11.77 MB PDF) [file pone.0003456.s003.pdf]
